# Comparison between metatranscriptomics and viral metagenomics, 16S, and host transcriptomics for comprehensive profiling of the respiratory microbiome and host response

**DOI:** 10.3389/fmicb.2025.1685035

**Published:** 2026-01-07

**Authors:** Gregory Destras, Marina Sabatier, Antonin Bal, Bruno Simon, Quentin Semanas, Hadrien Regue, Theophile Boyer, Dominique Ploin, Yves Gillet, Bruno Lina, Hussein Anani, Laurence Josset

**Affiliations:** 1 Laboratoire de Virologie, Centre National de Référence France-Sud des Virus des Infections Respiratoires, Plateforme de séquençage Genepii, Hospices Civils de Lyon, Groupement Hospitalier Nord, Lyon, France; 2 CIRI, Centre International de Recherche en Infectiologie, Team VirPath, Univ Lyon, Inserm, U1111, Université Claude Bernard Lyon 1, CNRS, UMR5308, ENS de Lyon, Lyon, France; 3 Hospices Civils de Lyon, Hôpital Femme Mère Enfant, Service de Réanimation Pédiatrique et d’Accueil des Urgences, Bron, France

**Keywords:** 16S gene sequencing, comparison, human transcriptomics, metatranscripomics, microbiome, multi-omics, viral metagenomics

## Abstract

**Introduction:**

Omics-based studies focusing on a single kingdom, such as bacterial 16S gene sequencing, viral metagenomics, and human mRNA sequencing, are commonly used to explore the microbiome and its association with host responses. But combining these approaches is often expensive and time-consuming. Metatranscriptomics provides a snapshot of the entire active microbiome through bulk RNA sequencing in a single test, yet its performance relative to kingdom-specific methods has not been systematically assessed.

**Methods:**

We compared metatranscriptomics with three kingdom-specific sequencing approaches in 20 nasopharyngeal aspirates from infants 7 months of age hospitalized for bronchiolitis at the Hospices Civils de Lyon.

**Results:**

Applying specific sequencing depth thresholds (≥1,000 bacterial reads, ≥100,000 human reads, and detection of an internal RNA control), metatranscriptomics showed high detection concordance and correlated abundance for RNA viruses and human coding genes. Metatranscriptomics also detected RNA from both eukaryotic and prokaryotic DNA viruses, suggesting potential for identifying transcriptional activity. For the bacteriome, 82% of genera exceeding 0.5% relative abundance were captured, revealing distinct transcriptional profiles at the species level. Metatranscriptomics reproduced multi-omics-derived host–microbiome endotypes and revealed stronger key microbial associations, particularly for transcriptionally active microorganisms.

**Discussion:**

These findings indicate that a single metatranscriptomics run can complement or replace kingdom-specific approaches for profiling RNA viruses and the host transcriptome, while also identifying transcriptionally active bacteria and DNA viruses. Low-abundance or latent microorganisms may still require targeted assays. Metatranscriptomics thus provides a cost- and time-efficient strategy for integrated microbiome research and holds promise for clinical applications in acute infections and cases of diagnostic uncertainty.

## Background

The term “microbiome” refers to the collection of all microorganisms and their genes present within a specific ecological niche. This includes viruses, known as the virome, bacteria, referred to as the bacteriome, and fungi, which constitute the mycobiome. The study of these microorganisms, their functions, and their links with host immune responses has gained significant interest over the last decade, leading to the discovery of new insights regarding health and pathologies (Liang and Bushman, 2021; Iorio et al., 2022).

Advances in sequencing methods have facilitated the direct exploration of microbial communities from clinical and environmental samples. In samples where microbial biomasses are not dominant, identifying all DNA microorganisms can be challenging. Shotgun metagenomics may lack sensitivity in identifying microorganisms in samples dominated by human DNA, so that strategies specifically trying to remove the human part and/or trying to specifically target the different components of the microbiome are required. For example, targeting genes encoding bacterial 16S ribosomal RNA or fungal internal transcribed spacers (ITS) DNA fragments has enabled the enrichment and identification of the taxonomic diversity of the bacteriome and the mycobiome of an ecological niche (Liang and Bushman, 2021; Iorio et al., 2022; Galloway-Peña and Hanson, 2020). Likewise, characterizing the virome requires specific metagenomics protocols, which frequently encompass viral enrichment procedures due to the absence of a distinctive genetic marker for viruses (Khan Mirzaei et al., 2021; Džunková et al., 2023). These sequencing approaches, which focus on a single kingdom, have proven to be valuable for exploring microbial communities, combating undocumented infections, identifying integrative microbiome signatures, and discovering cross-kingdom correlations associated with different health conditions (Batool and Galloway-Peña, 2023; Chiu and Miller, 2019; Zinter et al., 2024; Mick et al., 2023; Anani et al., 2025; Cao et al., 2024). However, given the differences in pretreatment steps, implementing all these independent approaches to comprehensively study how these different kingdoms articulate within the human microbiome can be labor-intensive, costly, and even limited when multiple sample extractions are needed, while the volume is limited.

Metatranscriptomics represents a promising all-in-one approach for identifying all active microorganisms present in a host immune response environment. In theory, metatranscriptomics could differentiate itself from the other, more kingdom-specific approaches by discriminating, in a single test, transcriptionally active DNA viruses and bacteria from latent microorganisms, while also capturing RNA viruses and host RNAs (Ojala et al., 2023). Some studies have carried out metatranscriptomics, mainly to decipher which of the putative pathways identified by metagenomics were transcriptionally active in the bacteriome, and to highlight transcriptionally correlated intra- and inter-kingdom microorganisms (Franzosa et al., 2018; Abu-Ali et al., 2018; Wang et al., 2023). In recent years, metatranscriptomics has also been performed for direct clinical applications, such as the identification of microorganisms not detected by routine diagnostics, and the determination of their resistance profile and their genotype (Ojala et al., 2023; Zhu et al., 2020; Rajagopala et al., 2021). In addition, some studies have reported that the integration of RNA-seq data from different kingdoms can reveal microbial signatures capable of distinguishing infectious from non-infectious states (Mick et al., 2023; Langelier et al., 2018; Kalantar et al., 2022; Zinter et al., 2021). Thus, metatranscriptomics offers the opportunity to achieve similar applications to targeted-kingdom approaches with two advantages: one single test and a comprehensive snapshot of the active microbiome. However, to our knowledge, no study has yet evaluated how metatranscriptomics can overlap or complement the results of strategies targeting a specific component of the microbiome.

This study aimed to address this gap by answering three main questions: (i) to what extent can metatranscriptomics replicate viral detection compared with viral metagenomics; (ii) how well does metatranscriptomics capture transcriptionally active bacteria compared with 16S gene profiling; and (iii) can metatranscriptomics reliably reflect the host transcriptional response compared with mRNA sequencing. Our working hypothesis is that metatranscriptomics can provide a single, comprehensive snapshot of the active microbiome and host response, potentially reducing the need for separate kingdom-specific assays. As such, comparison needs to be performed from clinical samples with frequent viral and bacterial coinfections. We leveraged a cohort of infants suffering from bronchiolitis, for which we previously performed kingdom-specific approaches to explore their microbiome (unpublished data).

## Materials and methods

### Sample collection

A clinical cohort of infants under 7 months of age who were hospitalized at the Hospices Civils de Lyon for bronchiolitis during the winters 2015–2017 was used for comparison. Initially, 53 samples were prospectively included for the analysis of the nasopharyngeal microbiome. The inclusion criteria were: (i) age <7 months, (ii) clinical symptoms of bronchiolitis requiring hospitalization, (iii) positive testing for Respiratory Syncytial Virus (RSV) or influenza virus, and (iv) no antibiotic exposure. Clinical and biological data, such as oxygen requirement, SpO_2_, respiratory rate, and length of hospital stay, were prospectively collected. Each nasopharyngeal sample was distributed into three 200-μL aliquots, including one with RNA later, and stored at −80 °C. All samples were subjected to three kingdom-specific methods: viral metagenomics sequencing (vir-mNGS), bacterial targeted sequencing (16S-Seq), and human mRNA targeted sequencing (mRNA-Seq). Among these, 29 were finally retained due to sufficient remaining material for metatranscriptomics (metaRNA-Seq).

### Viral metagenomics sequencing (vir-mNGS)

For each sample, one aliquot was thawed, and vir-mNGS was conducted in accordance with the previously described methodology (Sabatier et al., 2020). Ten no-template controls (NTCs) followed the same sample preparation as the samples. The first step was the incorporation into all samples of a single-stranded 2,800 bp RNA virus, MS2 *Escherichia* phage, used as the internal control (IC). MS2 was supplemented so that the concentration was set to an equivalent cycle threshold (Ct) of 38, which is close to the detection threshold of PCR assays. Before DNA/RNA extraction, viral particle enrichment steps consisted of centrifugation, filtration, and residual human and bacterial DNA removal by Turbo DNAse (Thermo Fischer) treatments. Extraction was performed on the Emag platform (Biomerieux) from 200 μL of specimen and 50 μL of elution. Elution was purified by acetate and linear acrylamide precipitation to obtain a 10 μL concentrated nucleic acid solution. Subsequent amplification was carried out by WTA2 (Thermo Fischer). Following library preparation by Nextera XT (Illumina), sequencing was finally performed on a 2 × 150 bp cartridge on Illumina Nextseq500 with a 20 pM final concentration and 1% of PhiX after a normalization step to ensure equal sequencing of each sample.

### Bacterial targeted sequencing (16S-Seq)

For each sample, one aliquot was thawed, and 16S-Seq sequencing was performed by the amplification of V1–V3 regions of 16S rRNA genes using an in-house protocol (Langevin et al., 2017) after nucleic acids extraction on the Emag platform (Biomerieux) from 200 μL of specimen and 50 μL elution. Seven no-template controls (NTCs) followed the same sample preparation as the samples. The sequencing was performed using a 2 × 300 bp cartridge on Illumina MiSeq with a 8 pM final concentration and 5% of PhiX.

### Human mRNA targeted sequencing (mRNA-Seq)

For each sample, 200 μL of the aliquot containing RNA later conservative was thawed, and the mRNA-Seq protocol was performed according to the NEBNext Single Cell/Low Input RNA library Kit for Illumina (NEB) protocol. Following human DNA removal by DNAseI treatment, RNA extraction was conducted on the RNEasy Plus Mini Kit (Qiagen), and amplification targeting polyadenylated RNAs was performed. Sequencing was performed on a 2 × 150 bp cartridge on Nextseq500 with a 20 pM final concentration and 1% of PhiX. The aliquot was again stored at −80 °C.

### Metatranscriptomics sequencing (metaRNA-Seq)

For each sample, 200 μL of the aliquot containing RNA later conservative, which was initially used for mRNA-seq, was rethawed. Three no-template controls (NTCs) followed the same sample preparation as the samples. Extraction and elution using 50 μL was conducted on a different protocol on the Maxwell RSC ADN Blood kit (Promega), but following the same initial step of MS2 supplementation of vir-mNGS. Subsequently, 8 μL of elution was subjected to DNase treatment and SPIA amplification using the Revelo kit following manufacturer recommendations (Tecan Diagnostic). Importantly, the Revelo kit is designed to avoid the amplification of ribosomal human RNAs. An automated process was performed on a Tecan Dreamprep FLUENT 780 with eight PCR cycles for library amplification, and normalized libraries were sequenced on a 2 × 100 bp SP cartridge on NovaSeq 6000 (Illumina) with a 400 pM final concentration and 1% of PhiX.

### Bacterial 16S-Seq bioinformatic process

All bioinformatics tools used are listed in Supplementary Table S1. The bacterial 16S genes were analyzed using the DADA2 R package (v3.2). The reads in all samples underwent trimming, filtering, and merging with the following parameters: trimLeft = 1 truncLen = c(298,280), maxN = 0, maxEE = c(2,5), truncQ = 2, rm.phix = TRUE, minOverlap = 10 maxMismatch = 0. This resulted in the generation of read counts for each bacterial Amplicon Sequence Variant (ASV) identified. The taxonomic assignment of these ASV was carried out using the silva_nr99_v138.1 database. Following the decontamination step described below, the read counts were aggregated to the genus level for the comparison.

### Viral metagenomics bioinformatics process

The trimming and filtering of raw paired reads was conducted using Cutadapt (minimum length of 30 bases, min quality of q20, and error rate set at 0), followed by a dehosting step (i.e., the removal of human reads) using SRAHumanScrubber from our in-house seqmet pipeline (scripts are available on https://github.com/genepii/seqmet). Duplicated reads were subsequently removed using bbmap. Taxonomic annotation of vir-mNGS was performed by Kraken2 with a threshold confidence value of 0.51, which was set to remove any false positive assignments. A custom database was used, such as viral taxonomy from RVDBv24.1^1^ and Inphared databases (v2 Nov2022, excluding phages with an unidentified host) (Cook et al., 2021), as well as archaeal, bacterial, fungal, and human taxonomy from RefSeq databases (downloaded with kraken2 21/09/2021). The read counts were aggregated to the family level for subsequent analyses.

### Human mRNA-targeted bioinformatics process

The raw paired reads were trimmed and filtered using Cutadapt, with a minimum length of 30 bases, a minimum quality of q20, and an error rate of 0. Gene annotation was performed using Hisat2 from the Rasflow pipeline with default parameters (Zhang and Jonassen, 2020) and the GRCh38 database. Duplicated reads were removed by using samtools, and the counts of RNAs mapping to the genes were performed using FeatureCounts. Only coding genes were retained for the comparison.

### Metatranscriptomics bioinformatic process

For the analysis of viral reads from metatranscriptomics, Cutadapt and SRAHumanScrubber were used for the preprocessing, quality assessment, and dehosting of the raw paired reads. The duplicated reads were removed by using bbmap, and the taxonomic annotation was performed using Kraken2 (confidence 0.51). The read counts were aggregated to the family level for subsequent analyses.

The same bioinformatic process was performed for bacterial metatranscriptomics, with the exception of two additional steps. The Revelo kit includes an SPIA step, avoiding human rRNAs amplification. Given the lack of knowledge regarding the impact on bacterial 16S rRNAs, we preferred to remove them from the dataset bioinformatically and to focus the comparison on the transcripts of bacteria for comparison. An additional step corresponding to a re-estimation of bacterial species abundance using the Bracken tool on Kraken2 reads was carried out with the default threshold (10 reads). The read counts were then aggregated to the genus level. Of note, Bracken was not used for abundance re-estimation of viral species abundances due to gaps in the viral taxonomy.

The process of analyzing mRNAs from metatranscriptomics was identical to that used for mRNA-Seq, with trimming/filtering and gene annotation performed by Cutadapt and Hisat2, respectively. Duplicated reads were removed with Samtools prior to obtaining RNA counts using FeatureCounts. Only coding genes were retained for the comparison.

### Decontamination

Read counts tables were normalized to RPM (read per million) in order to identify bacterial (at the ASV or genus level) and viral (at the family level) contaminants. First, a comparison of the prevalence of positive taxons in samples and NTCs was carried out using the decontam R package (v1.20.0). Second, all putative bacterial and viral contaminants identified with a mean RPM value five times higher in NTCs than in samples were removed. A summary of contaminants for each protocol is provided in Supplementary Table S2. In addition, we removed all Proteus-phage *VB*-PmiS-*Isfahan* identifications, which we previously identified as a false positive signal from Kraken2 (internal data).

### Sequencing quality controls and specific normalization for each kingdom comparison

All samples that encountered our initial quality controls were selected for analysis. These included the presence of at least one read of MS2 IC in vir-mNGS and metaRNA-Seq results, at least 10,000 mapped reads, a reached rarefaction plateau for 16S-Seq, and at least 10,000 mapped reads for mRNA-Seq.

For the comparison between metaRNA-Seq and each kingdom-specific approach, the normalization process was conducted in a specific manner. The comparison of viral families was conducted by taking advantage of the presence of MS2 IC in the two datasets. The RPM counts of each viral family were transformed by its additive log-ratio (ALR), defined as log10 (RPM viral family x/RPM IC MS2) (Greenacre et al., 2021). To improve graphical visualization and distinguish between families with and without reads, a pseudocount of 10^−5^ was arbitrarily added to the viral family/MS2 ratio. This means that families with no reads were assigned an ALR of −5. Bacterial genera identified by 16S-Seq and metaRNA-Seq were compared based on their relative abundances. The comparison of human coding genes was conducted by a log_2_(RPM) normalization.

No batch correction was performed as all samples belonged to the same batch for metatranscriptomics and for kingdom-specific omics.

### Multi-omics integration

Microbial count matrices from each kingdom-specific omics dataset and from metaRNA-Seq were used in parallel, all normalized by *z*-score, to perform multi-omics integration using Multi-Omics Factor Analysis (MOFA2 R package v.1.12.1). To reduce inter-patients variability, viral families and bacterial genera were filtered using a prevalence threshold of 30% across samples, and host transcripts were filtered at 50%. For the metaRNA-Seq dataset, bacterial species were included as an additional independent view. Aggregated diversity metrics (Shannon and richness indexes) were also incorporated into bacterial and viral count matrices to form final comprehensive count matrices, representing independent bacterial, viral, and human view components in both the multi-omics and metaRNA-Seq datasets.

To capture the full variance explained by the model, all latent factors were retained (15 factors for the multi-omics analysis and 10 factors for the metatranscriptomics analysis). The latent factors were then used as input for Uniform Manifold Approximation and Projection (umap R package v.0.2.10.0) with the following parameters: *n_neighbors = 5, min_dist = 0, n_components = 2*. The optimal number of clusters was determined using gap statistic, silhouette width, and total within sum of square method, followed by k-means (stats R v4.3.1) clustering with parameters: *centers = 4, nstart = 2.* Functional enrichment of each cluster was determined according to the compareCluster function of the clusterProfiler R package (v.4.10.1).

### Statistics

Overall concordance between the two techniques was evaluated based on the total rate of co-detection of viral families, bacterial genera, or transcripts across all samples. For bacterial concordance, we analyzed all co-detections between the datasets as well as co-detections exceeding defined relative abundance thresholds of 0.5 and 10% in order to evaluate concordance for bacteria with low versus high relative abundances detected in 16S-Seq. Statistical tests and figures were performed with RStudio (v4.1.6) by using the ggplot2 package (v3.4.2). Median comparisons were performed using Mann–Whitney *U* test or Kruskal–Wallis tests, while mean comparisons were performed with a *t*-test. Proportion comparisons were performed using Fisher’s exact test. No multiple-testing correction was applied because the distribution of *p*-values across comparisons did not meet the assumptions required for standard FDR adjustment. Correlations were carried out with Pearson tests. Enrichment of genes coding for antiviral defense (GO:0051607) was performed using the biomaRt R package (v3.18). Effect sizes were computed with the vegan R package (2.6).

## Results

### Metatranscriptomics (metaRNA-Seq) allowed visualizing the global repartition of each active kingdom

**Figure 1:**
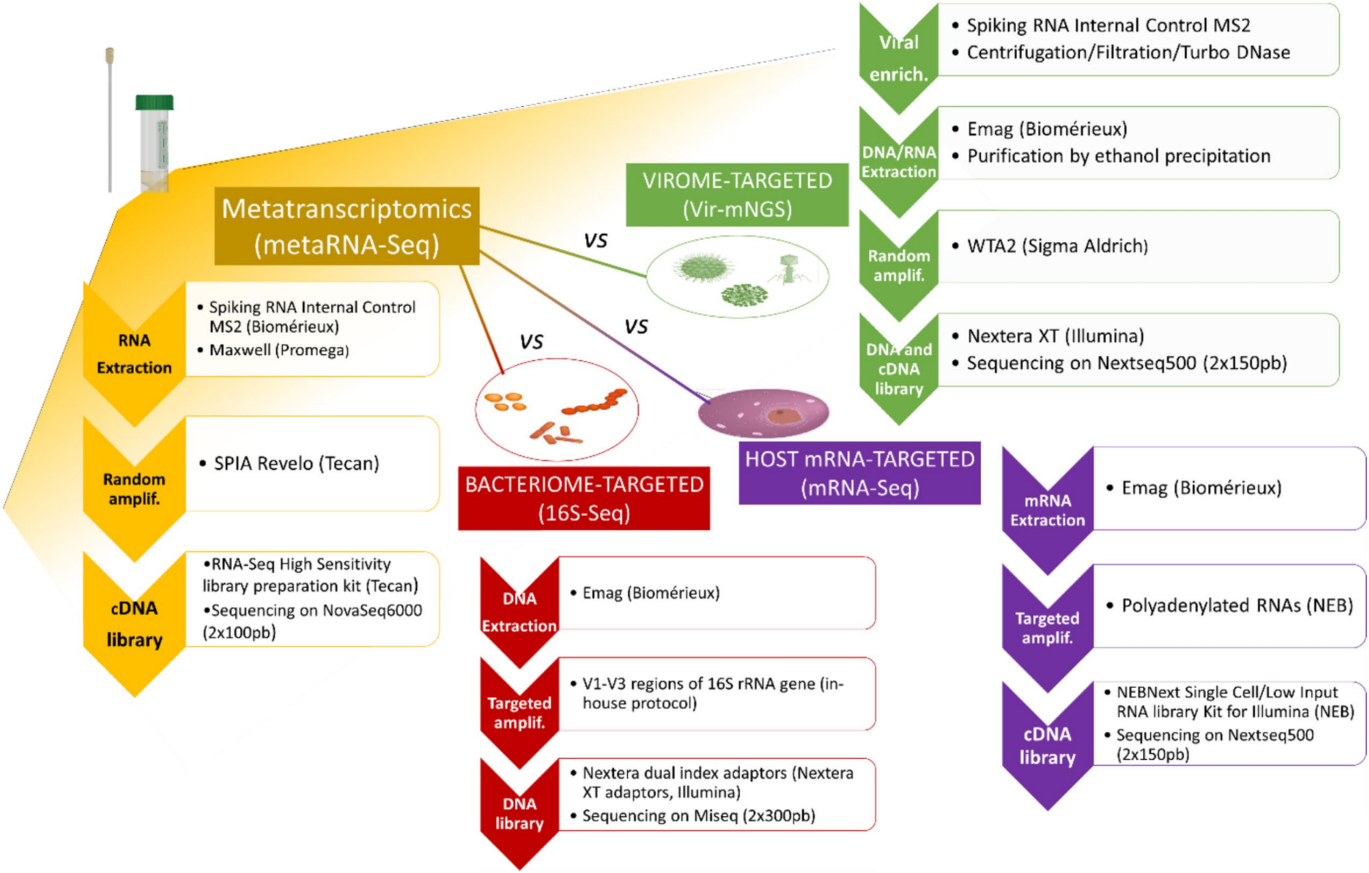
Sample preparation workflows used for metatranscriptomics and kingdom-specific sequencing approaches.

**Figure 2:**
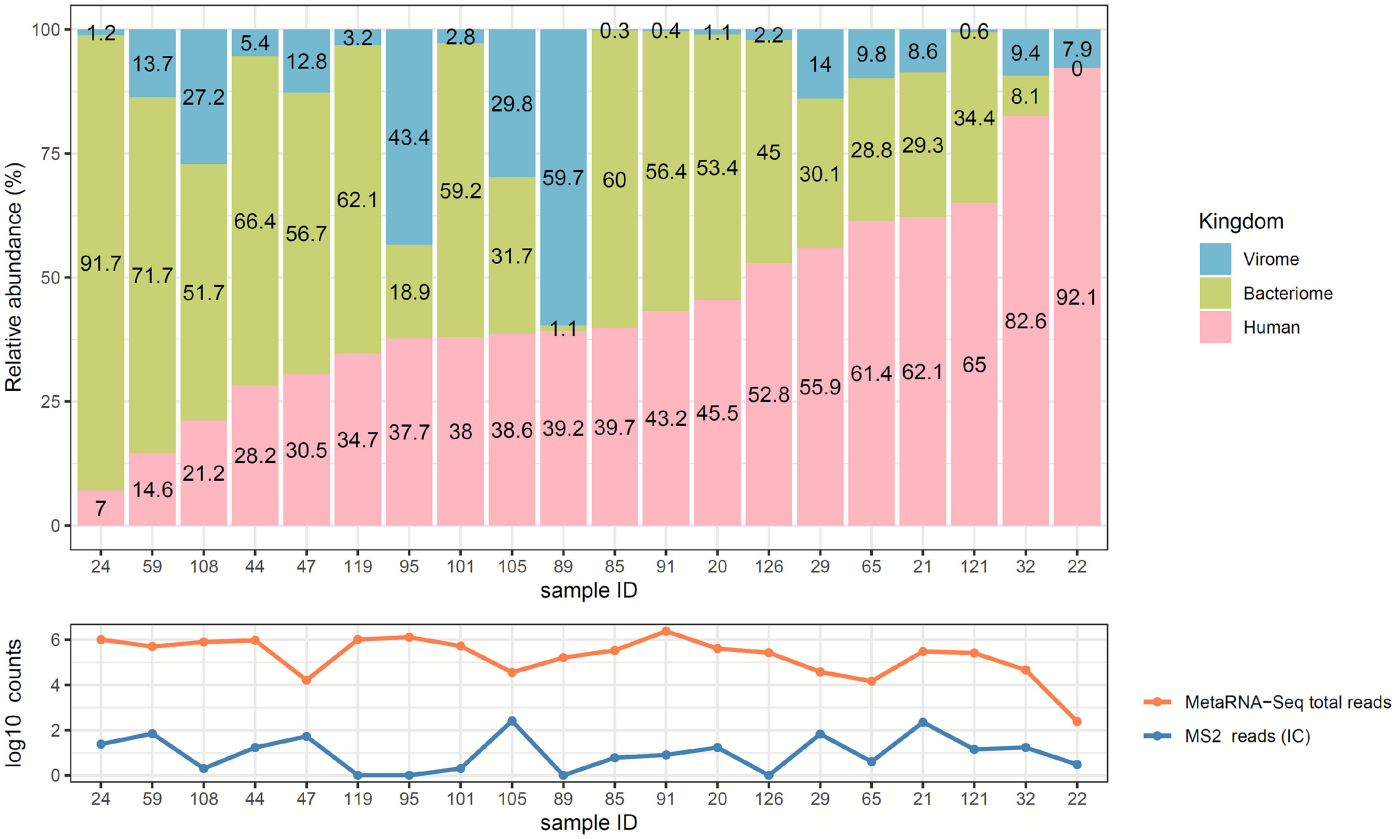
Distribution of kingdoms in metatranscriptomics (metaRNA-Seq). The upper panel represents the relative abundances of the different microbiome layers (virome in blue, bacteriome in green, and human in pink) for the 20 samples. The lower panel shows the log10 counts of metaRNA-Seq total reads (orange) and internal control (IC) reads (blue).

A total of 29 nasopharyngeal samples were sequenced with the four techniques, such as the three kingdom-specific sequencing methods, viral metagenomics (vir-mNGS), bacterial 16S gene profiling (16S-Seq), human mRNA sequencing (mRNA-Seq), and metatranscriptomics (metaRNA-Seq) (Figure 1). Each kingdom-specific sequencing method was considered as a gold standard for subsequent comparisons. To ensure a suitable comparison, only samples meeting all initial QC criteria for the kingdom-specific sequencing approaches were retained: (1) ≥ 1 IC read for vir-mNGS; (2) ≥ 10,000 mapped reads and a reached rarefaction plateau for 16S-Seq; and (3) ≥ 10,000 mapped reads for mRNA-Seq. For metaRNA-Seq, no initial QC filter was applied, except for the detection of ≥1 IC read, resulting in a final set of 20 samples meeting all required QCs. The clinical characteristics and biological results of the 20 selected patients for the comparison are detailed in Supplementary Table S3. Sequencing characteristics for the samples and NTCs are summarized in Supplementary Tables S4, S5, respectively. MetaRNA-Seq results are further described in Figure 2 and below. Overall, the sequencing depth obtained with MetaRNA-Seq ranged from 0.1 M to 19.2 M raw reads, with a median of 7.2 M reads. One sample was particularly poorly sequenced (#22), but was retained for subsequent analysis as IC (MS2 phage) was detected. IC read counts ranged between 1 to 63 reads, which represented 1 to 4,132 RPM interpretable reads of the total metatranscriptome (Figure 2, Supplementary Table S4). IC detection was not associated with metaRNA-Seq sequencing depth (*p* = 0.13) nor virome relative abundance (*p* = 1.0).

The median relative abundances of the viral, bacterial, and human parts within their metatranscriptome represented 8.2, 48.3 and 39.5%, respectively (Figure 2). However, there was considerable variability in the abundance of each kingdom within its metatranscriptome. The viral part represented from 0.3 to 60% of the metatranscriptome, while the bacterial and human parts accounted for 1 to 92% and 7 to 92%, respectively. In our comparison, two patterns stand out: one with a predominantly bacterial part (10/20 samples) and the other with a predominantly human part (7/20 samples). Another single sample had a predominantly viral part (#89), while samples #105 and #95 were evenly distributed.

#### Overview of metaRNA-Seq performance

We evaluated the ability of metaRNA-Seq to capture the same biological signals as kingdom-specific sequencing approaches (vir-mNGS, bacterial 16S-Seq, and human mRNA-Seq) in terms of detection and abundance (Table 1). For RNA viruses, metaRNA-Seq captured 86% of vir-mNGS detections, with strongly correlated abundance estimates (Pearson *r* = 0.83), and also detected 32% of DNA viruses identified by vir-mNGS. For bacteria, 82% of genera exceeding 0.5% relative abundance in 16S-Seq were detected, with relative abundances correlated for some taxa, though notable differences between DNA and RNA profiles were observed. For the host transcriptome, 78% of coding genes identified by mRNA-Seq were captured, with moderate overall correlation (*r* = 0.56) and stronger correlation for antiviral response genes (*r* = 0.79). Overall, metaRNA-Seq reproduced key features of RNA-based kingdom-specific methods (RNA viruses and host transcriptome) and partially captured DNA microbiome components (bacteria and DNA viruses). Detailed results for each kingdom are presented below.

**Table 1:** Overview of metaRNA-Seq performances.

Kingdom	Comparison method	Detection concordance	Abundance correlation	Normalization/thresholds	Key notes
RNA viruses	vir-mNGS	86% of detections captured	Pearson *r* = 0.83	Additive log-ratio (ALR) using MS2 internal control	Captured co-infections; RNA viruses reliably quantified
DNA viruses	vir-mNGS	32% of detections captured	Not significantly correlated (Pearson *r* = 0.22, *p* = 0.19)	ALR using MS2 internal control	Partial capture suggests transcriptional activity of DNA viruses
Bacteria	16S-Seq	82% of genera >0.5% RA detected	Correlated for some genera; differences observed between DNA/RNA	Relative abundance thresholds: 0.5 and 10%	Species-level resolution achieved; transcriptional activity differs across genera
Host transcriptome	mRNA-Seq	78% of coding genes captured	Moderate overall (*r* = 0.56); antiviral genes stronger (r = 0.79)	Log₂RPM	Adequate capture of immune signatures; high sequencing depth required

RA, relative abundance; ALR, additive log-ratio normalization using MS2 internal control for viral RNA.

Thresholds for viral, bacterial, and host detection ensure reliable interpretation (≥1,000 bacterial reads, ≥100,000 human reads).

### MetaRNA-Seq identifies RNA from both eukaryotic and prokaryotic DNA viruses

Normalization of read expression for both vir-mNGS and viral metaRNA-Seq was conducted by using an additive log-ratio (ALR) transformation, with the MS2 IC (spiked at a concentration equivalent to a Ct of 38 for a virus of 2,800 pb) used as the denominator. An ALR value above 0 indicated viral abundance higher than IC. A positive threshold of the gold standard vir-mNGS was set as ALR ≥ −1, thus considering all detections equivalent to a Ct above 41. Below, the detection of RNA and DNA viruses by metaRNA-Seq is described separately.

**Figure 3:**
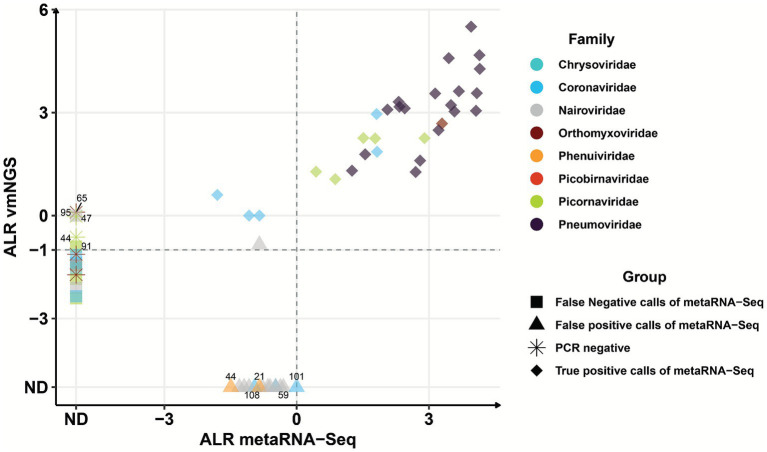
Comparison of RNA viral families hits identified by viral metagenomics (vir-mNGS) and metatranscriptomics (metaRNA-Seq). Expression of all RNA viruses detected in the 20 patients is represented according to their additive log-ratio (ALR) with MS2 IC as reference denominator (i.e., an ALR of 0 indicates that a viral family is detected at the same number of reads as MS2 IC). Each color represents a viral family. Concordant hits in the two techniques are represented with a diamond, while specific and missed calls in metaRNA-Seq are shown as triangles and squares, respectively. Samples positive in vir-mNGS but tested negative by PCR are indicated with stars. The horizontal dashed line corresponds to the viral metagenomics (vir-mNGS) threshold of −1 ALR (i.e., number of reads for a 10 times lesser than IC). The vertical dashed line represents the threshold of comfortable interpretation for metaRNA-Seq. Numbers refer to sample IDs discussed in the Results section.

Considering the threshold, viral metaRNA-Seq demonstrated a sensitivity of 86.1% (31/36) and a Jaccard index of 0.86 relative to vir-mNGS, identifying the majority of RNA viruses detected by the latter (Figure 3). True detections included 19 *Pneumoviridae* (ALR 1.2–4.1) and 1 *Orthomyxoviridae* (ALR 3.3), in concordance with RT-PCR routine testing (Supplementary Table S3). Additionally, five hits of both *Picornaviridae* (0.4–2.9 ALR) and *Coronaviridae* (−1.8–1.9 ALR) were detected by the two techniques as co-infections of RSV (belonging to *Pneumoviridae*) in nine samples. Importantly, ALR values for RNA viruses were highly correlated between the two techniques (*p* < 0.001, Pearson *r* = 0.83) (Supplementary Figure S1). In addition, *Pneumoviridae* ALR’s from metaRNA-Seq were inversely correlated with RSV Ct values from RT-PCR (Pearson *r* = −0.6, *p* = 0.03) (Supplementary Figure S2).

Five viruses detected by vir-mNGS were missed by metaRNA-Seq, corresponding to a missed rate of 13.9% (5/36). These included one *Orthomyxoviridae* (#65), three *Picornaviridae* (#44, #47, #91), and one *Nairoviridae* (#95). All displayed negative ALR values, indicating very low abundance. RT-PCR data were available for three of these samples (Supplementary Table S3), all negative, suggesting possible specific calls from vir-mNGS, likely due to manual handling contamination. To assess potential specific calls of metaRNA-Seq, we examined the 14 RNA viral hits exclusively detected by this technique. All of these detections showed ALR values ≤ 0. These included detections of *Phenuiviridae* (samples #44 and #21) and *Coronaviridae* (#108, #59, and #101) (Figure 3). Additionally, we noted that viruses from the *Nairoviridae* family were detected by both techniques in only 1/12 (8%) samples, while they exclusively appeared in metaRNA-Seq in 10/12 (83%) samples.

Altogether, this first comparison led to the point that for RNA viruses, positive ALRs in metaRNA-Seq can be considered as concordant, while negative ALRs should be interpreted with caution, with confirmation required by RT-PCR.

**Figure 4:**
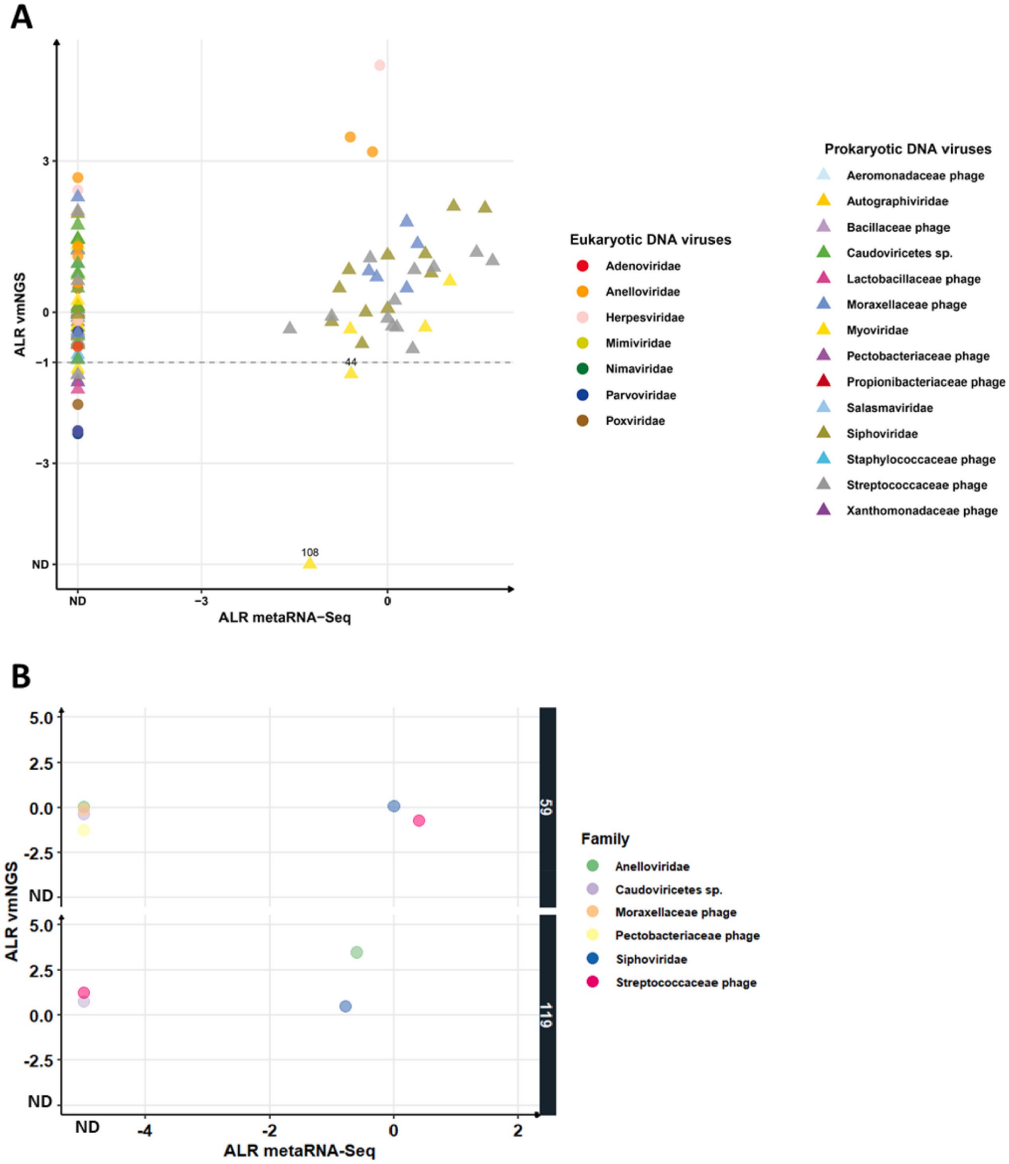
Comparison of DNA viral families identified by viral metagenomics (vmNGS) and metatranscriptomics (metaRNA-Seq). Expression of all DNA viral families identified in the 20 patients **(A)** and specifically in two patients **(B)** is represented according to their additive log-ratio (ALR) with MS2 IC as reference denominator (i.e., an ALR of 0 indicates that a viral family is detected at the same number of reads as MS2 IC). Each color represents a viral family. Eukaryotic or prokaryotic viruses are represented by circles and triangles, respectively. The dashed line corresponds to the vmNGS threshold of −1 ALR (i.e., number of reads for a species 10 times lesser than IC). Numbers refer to sample IDs discussed in the results section.

To evaluate the detection of DNA viruses by metaRNA-Seq, we applied the same positivity threshold (ALR ≥ −1) used for vir-mNGS. Overall, metaRNA-Seq identified 34 out of 95 DNA viruses (35.8%) detected by vir-mNGS, with ALR values ranging from −1.6 to 1.7. ALRs were not significantly correlated with those from vir-mNGS (Pearson *r* = 0.22, *p* = 0.19) (Figure 4A).

Among eukaryotic DNA viruses, metaRNA-Seq detected only 3 out of 19 (15.8%): one Herpesvirus in sample #24 (ALR − 0.2 vs. 4.9 in vir-mNGS), two Anelloviruses in samples #119 and #21 (ALRs −0.6 and −0.2 vs. 3.5 and 3.2, respectively). For prokaryotic DNA viruses, metaRNA-Seq identified RNA in 31 out of 76 (41%) cases, with ALRs spanning −1.6 to 1.7. These detections included *Moraxella* Caudovirales phages (5/11 samples), *Streptococcus* phages (12/17), *Siphoviridae* (11/18), and *Myoviridae* (3/6).

No significant difference was observed in vir-mNGS ALR averages between prokaryotic viruses detected or undetected by metaRNA-Seq (*p* = 0.72), indicating that detection may be influenced more by transcriptional activity than overall abundance. Notably, only 2/34 DNA viral hits (5.9%) were exclusively detected by metaRNA-Seq, both *Myoviridae*: one with ALR-1.2 (just below vir-mNGS threshold) in sample #44 and one with ALR-1.3 in sample #108, which showed higher sequencing depth in metaRNA-Seq than in vir-mNGS (Supplementary Table S4).

Interestingly, we noted that among different patients, the profiles of DNA viruses detected by both vir-mNGS and metaRNA-Seq were not identical. As illustrated in Figure 4B, RNA from Siphoviridae was detected in both patients, whereas RNA from *Streptococcaceae* phage was found in patient #59 but not in #119, while RNA from *Anelloviridae* showed the opposite pattern.

These findings suggest that metaRNA-Seq offers the ability to detect RNA from eukaryotic and prokaryotic DNA viruses, with rare exclusive detections that may reflect low-level transcription or sequencing depth biases.

### MetaRNA-Seq took a snapshot of highly transcriptionally active bacteria with species identification

**Figure 5:**
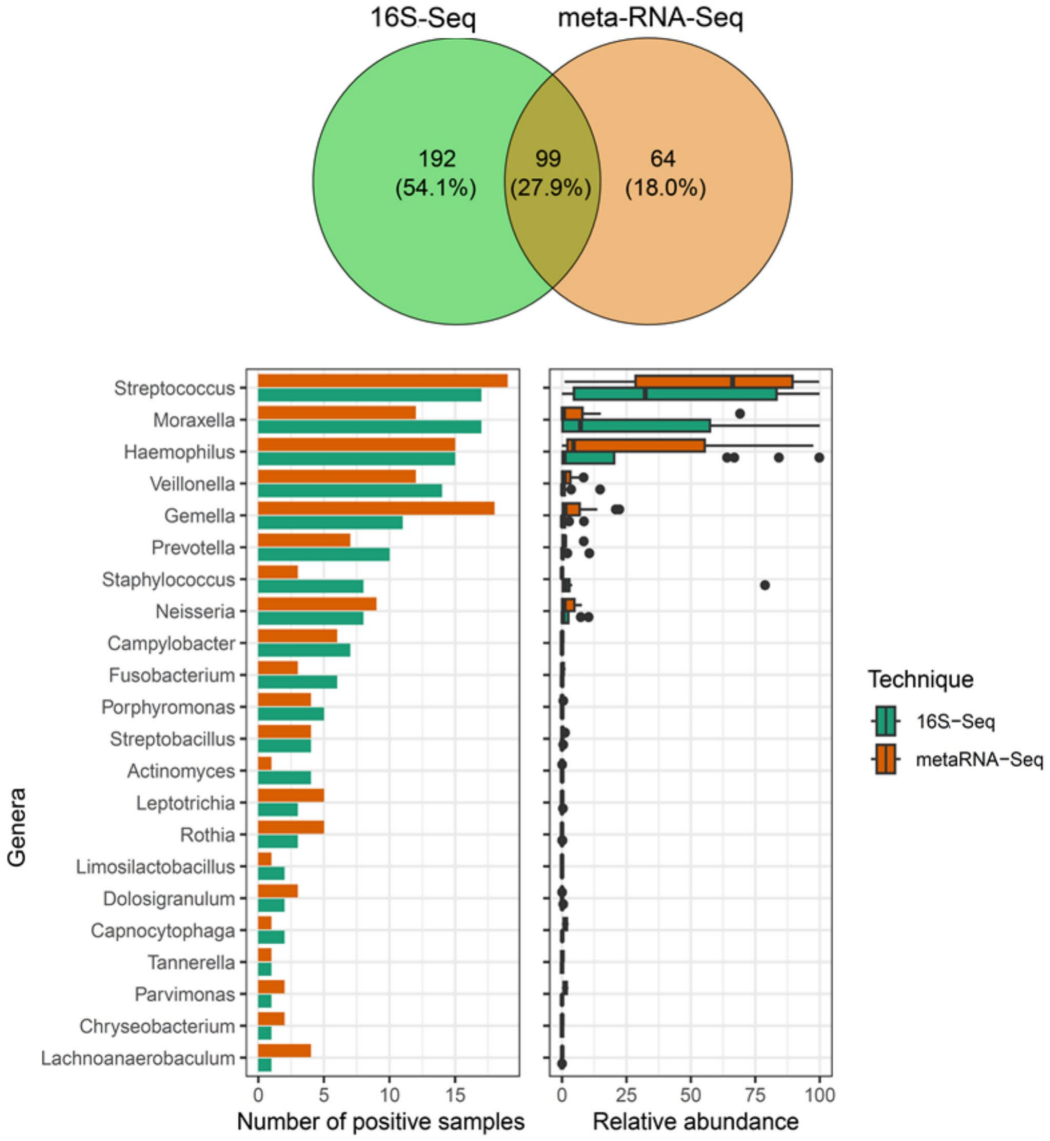
Comparison of bacterial genera detected by 16S-Seq and metatranscriptomics (metaRNA-Seq). The upper panel represents the Venn diagram for all bacterial genera hits detected across the 19 samples meeting the QC threshold of at least 10,000 reads by 16S-Seq (green), metaRNA-Seq (orange), or both (dark yellow). Bar plots (left panel) indicate the number of positive samples, and box plots (right panel) show the corresponding distribution of relative abundances, with medians represented, for bacterial genera identified by both approaches.

To assess the concordance between 16S rRNA gene sequencing (16S-Seq) and metatranscriptomic profiling (metaRNA-Seq), we compared the relative abundances of bacterial genera identified by each technique. Sample #22 was excluded from analysis due to insufficient sequencing depth in metaRNA-Seq. Compared to all other samples, this one presented fewer than 1,000 reads mapping to bacterial reads and a rarefaction curve that failed to reach a plateau. Across all 19 samples finally included, 99 out of 355 bacterial genus-level hits (27.9%) were detected by both methods. Specifically, 35.6% (99/291) of all bacterial genera identified by 16S-Seq were also found to be transcriptionally active by metaRNA-Seq (Figure 5). The most frequently co-detected genera, *Streptococcus, Moraxella, Haemophilus, Veillonella, and Gemella*, were also among the most abundant in both the 16S-defined bacteriome and the metatranscriptome.

To estimate the sensitivity of metaRNA-Seq for capturing abundant taxa, we examined RNA detection among bacterial genera with two different relative abundance thresholds. Among genera exceeding 0.5 and 10% relative abundance in 16S-Seq, 82.4% (56/68) and 96.4% (27/28), respectively, were also detected by metaRNA-Seq. This high detection rate for abundant taxa supports the sensitivity of metaRNA-Seq for identifying transcriptionally active bacteria. However, four genera, *Actinobacillus* (0.003–10.0%), *Bergeyella* (0.002–0.3%), *Alloprevotella* (0.005–2.0%), and *Granulicatella* (0.002–0.1%), were identified in ≥5 samples by 16S-Seq but never detected as transcriptionally active, potentially reflecting bacteria with low, silent, or absent transcriptional activity (Supplementary Figure S3).

To evaluate specificity, we focused on bacterial genera exclusively detected by metaRNA-Seq. We identified 64 such hits, of which only 11 exceeded 0.5% relative abundance (Supplementary Figure S4). Among these, several genera were found in multiple samples (0.8–6.6% relative abundances), such as *Bifidobacterium, Schaalia, Candidatus Nasosynobacter, Pseudoprevotella, Enterococcus, Histophilus, Phocaeicola, and Grimontia*. Other isolated exclusive detections involved *Streptococcus*, *Haemophilus*, and *Gemella* (relative abundances: 0.5–30%), which may reflect true signals rather than noise.

**Figure 6:**
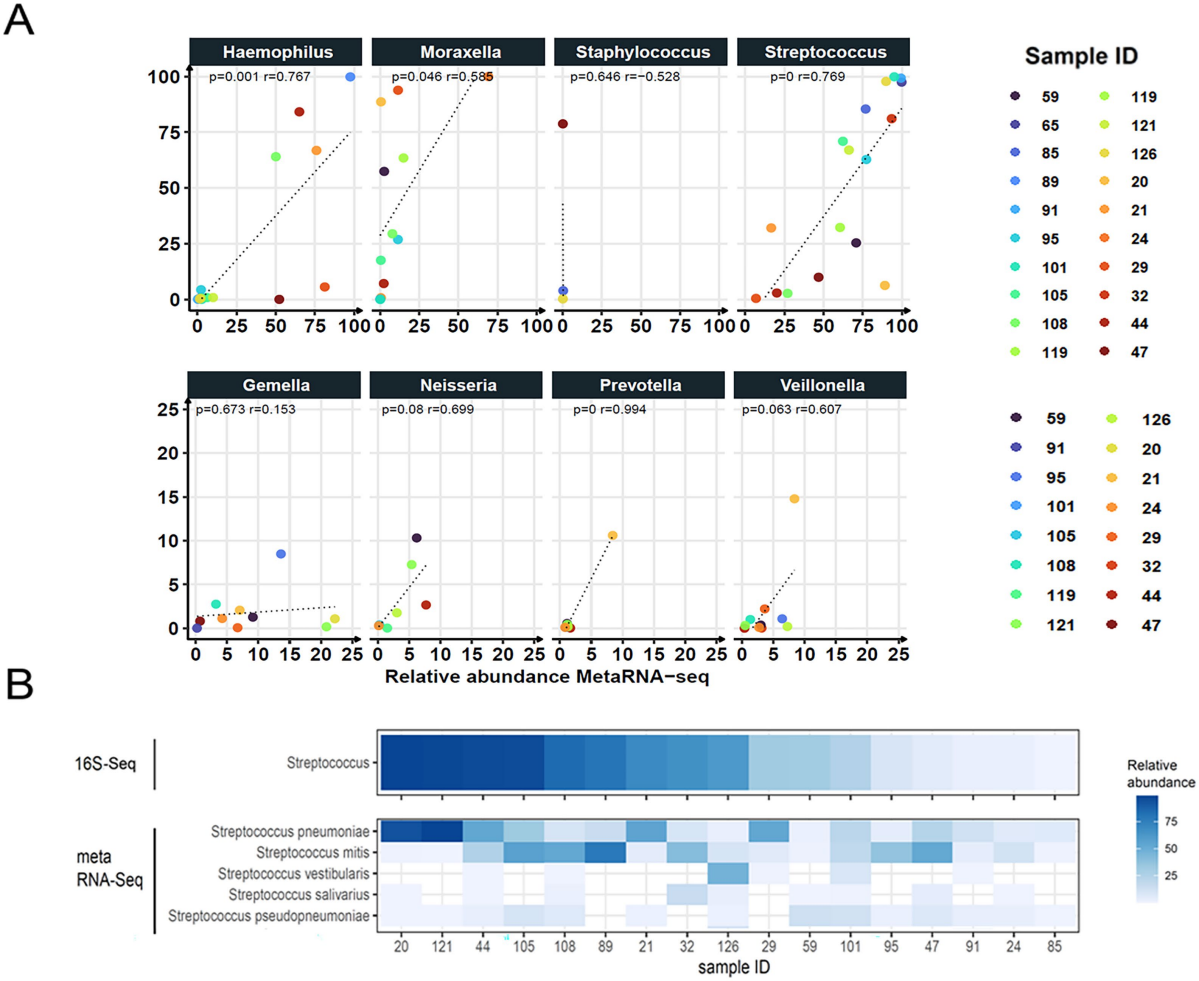
Representativity of bacterial genera for 16S-Seq and metatranscriptomics (metaRNA-Seq). **(A)** The top eight bacterial genera are represented according to their relative abundances in both techniques. Each color corresponds to a sample ID. The dashed line indicates the regression line, and the Pearson correlation test was used to calculate *p*-values and correlation coefficients between relative abundances in 16S-Seq and metaRNA-Seq. **(B)** Relative abundances of the top five *Streptococcus* species identified by metaRNA-Seq are depicted against the relative abundance of *Streptococcus* genera in 16S-Seq.

We then assessed whether the relative abundance profiles of bacterial genera were similar between the two methods. *Streptococcus, Moraxella, Prevotella, and Haemophilus* showed significant Pearson correlations between taxonomic presence (16S-Seq) and transcriptional activity (metaRNA-Seq) (Figure 6A). Interestingly, while *Streptococcus* displayed a regression pattern suggesting equivalent abundance and transcriptional activity, other genera, such as *Moraxella* and *Staphylococcus*, showed high relative abundance by 16S-Seq but very low transcriptional activity. This suggests genus-specific differences in metabolic activity. Likewise, species-level variability in activity may account for outlier points along the regression lines. Actually, only 4% of bacterial taxa were assigned to genus level by 16S-Seq, whereas metaRNA-Seq enabled species-level resolution of transcriptionally active taxa. This was exemplified by *Streptococcus* species. Although samples #126, #32, and #21 showed similar relative abundances in 16S-Seq, metaRNA-Seq revealed distinct dominant species: *S. vestibularis* in sample #126, *S. mitis* in #32, and *S. pneumoniae* in #21 (Figure 6B). This underscores the added value of metatranscriptomics in uncovering functional diversity and identifying actively transcribing microbial populations beyond taxonomic presence.

### Antiviral host-response transcriptional activity was concordant between mRNA-Seq and metaRNA-Seq

**Figure 7:**
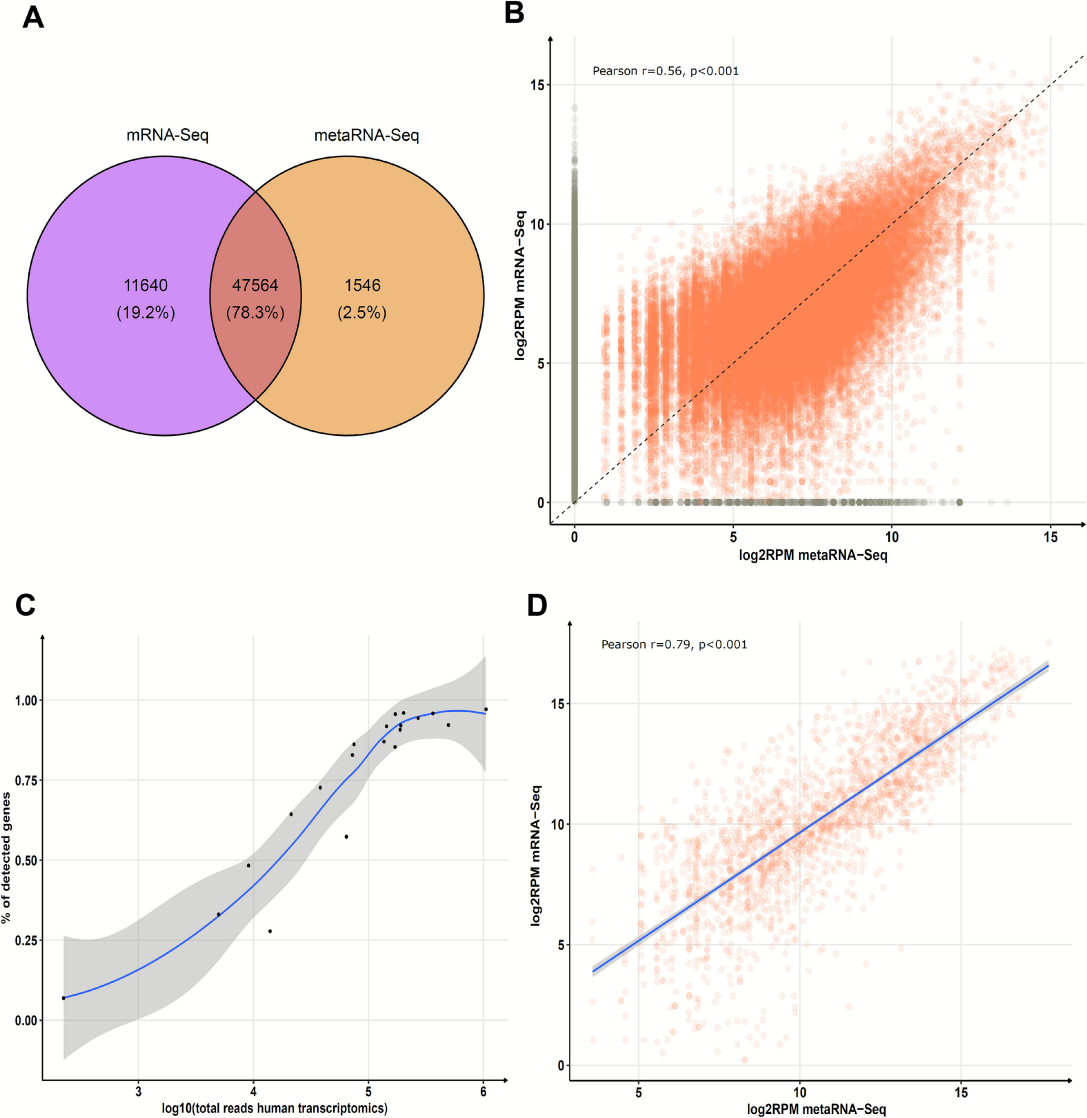
Comparison of human transcripts between capture-based human transcriptomics (mRNA-Seq) and metatranscriptomics (metaRNA-Seq). **(A)** Venn diagram showing human mRNA hits for genes present in more than 85% of the 20 samples by mRNA-Seq. **(B)** Distribution of human mRNA hits for genes present in more than 85% of the 20 samples by mRNA-Seq. Transcripts are normalized as Log_2_ Read per million (RPM). Colors indicate mRNA hits identified by both techniques (orange) or solely by one technique (gray). The dashed line represents the theoretical perfect correlation. **(C)** Proportion of expected human genes detected by metaRNA-Seq relative to mRNA-Seq, plotted against sequencing depth (total human reads per sample). **(D)** Distribution of human mRNA hits for genes involved in antiviral defense for the 11 samples meeting the QC threshold of at least 5 log_10_ human reads. The blue line corresponds to the regression curve.

To evaluate the performance of metaRNA-Seq in profiling human gene expression, we restricted our comparison to protein-coding genes consistently detected by mRNA-Seq in at least 85% of samples (17/20). Across all transcripts, we observed a global concordance of 47,564 out of 60,751 transcripts (0.78 Jaccard index), corresponding to 3,246 unique genes, among which 3,240 (99.8%) were also detected by metaRNA-Seq (Figures 7A,B). Expression levels (log₂RPM) were significantly correlated between metaRNA-Seq and mRNA-Seq across these genes (Pearson *r* = 0.56, *p* < 0.001), confirming a moderate but robust consistency in expression quantification (Figure 7B).

Only six genes, *CDK11A, PRRG3, NDUFA13, HBA2, TMA7B*, and *UBE2L5*, were missed by metaRNA-Seq. These genes showed significantly lower expression in mRNA-Seq compared to the other genes (mean log₂RPM: 2.4 vs. 6.2, *p* < 0.001), indicating that low expression is a limiting factor for detection by metaRNA-Seq. More broadly, the set of transcripts missed by metaRNA-Seq also exhibited lower average expression than those detected (mean log₂RPM: 5.3 vs. 6.5, *p* < 0.001), confirming a sensitivity threshold linked to transcript abundance.

To establish a quality threshold for reliable interpretation of host gene expression, we analyzed the impact of human read depth in metaRNA-Seq on gene detection. We found that a plateau in detection rate was reached at ≥100,000 total human reads per sample (5 log₁₀ reads) (Figure 7C). Based on this criterion, we retained 11 samples with sufficient depth for downstream transcriptome analyses. Within this subset, we tested whether metaRNA-Seq could accurately capture host immune signatures. Focusing on the 216 genes involved in antiviral defense (GO:0051607), we observed a strong correlation in expression levels between metaRNA-Seq and mRNA-Seq (*r* = 0.79, *p* < 0.001) (Figure 7D). These results demonstrate that when sequencing depth is adequate, metaRNA-Seq provides high specificity and sensitivity for profiling host response, particularly antiviral gene expression.

### MetaRNA-Seq and multi-omics reveal similar endotypes with enhanced microbial resolution from metaRNA-Seq

**Figure 8:**
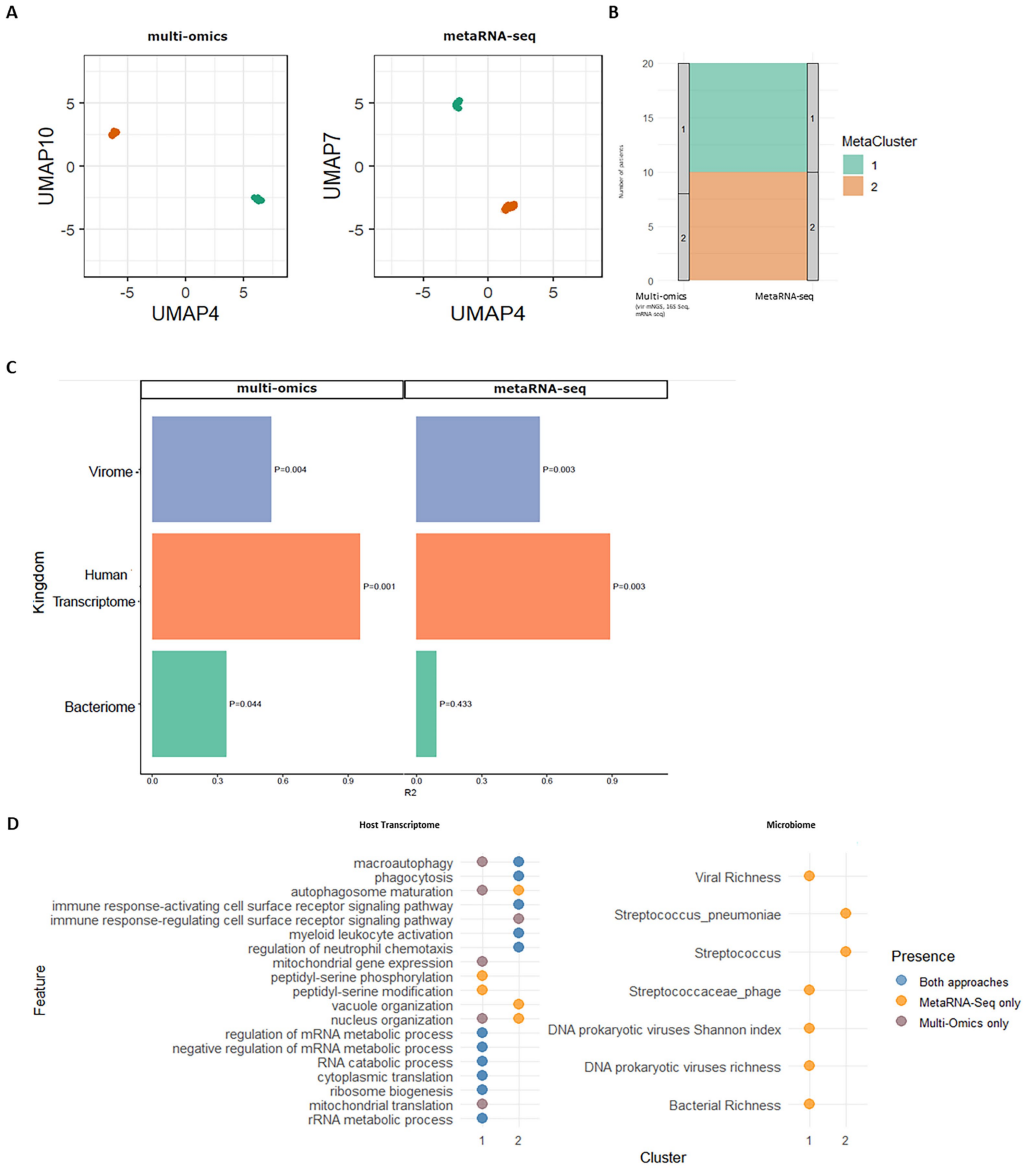
Characterization of microbial-host endotypes identified by integration of either kingdom-specific multi-omics or metatranscriptomics (metaRNA-Seq). **(A)** *K*-means clustering applied to Uniform Manifold Approximation and Projection (UMAP) plots based on latent factors derived from Multi-Omics Factor Analysis (MOFA), generated using either kingdom-specific (multi-omics) sequencing data (left panel) or metatranscriptomics (metaRNA-Seq) data (right panel). **(B)** Alluvial plot showing the distribution of same patients across clusters, comparing their assignments between the two datasets. **(C)** Effect sizes of microbial and host compositional layers contributing to endotype characterization for multi-omics and metaRNA-Seq approaches **(D)** Significant host pathways and microbiome features associated with each cluster according to both approaches. Features common to both approaches are shown in blue; those specific to kingdom-specific methods in grey, and those unique to metaRNA-Seq are in gold.

Beyond analyzing virome, bacteriome, and host-response separately, a key strength of integrated omics is the ability to identify co-associated features within samples, revealing meaningful microbiome–host endotypes. To evaluate the capacity of metaRNA-Seq to recapitulate the clustering structure obtained by multi-omics integration, we performed unsupervised clustering independently using either the multi-omics dataset or metaRNA-Seq data alone (Figure 8A). For both approaches, we created equivalent input matrices by *z*-scoring the counts of bacterial genera, viral families, and host transcripts. In addition, Shannon diversity and richness indices were incorporated for both datasets, and bacterial species were added only to the metaRNA-Seq dataset. A Multi-Omics Factor Analysis (MOFA) was applied to each dataset (Supplementary Figure S5), and the latent factors were projected using 2D Uniform Manifold Approximation and Projection (UMAP). Clustering based on the resulting latent factors revealed the robust presence of two distinct endotypes in both datasets, supported by multiple hierarchical clustering indices (Figure 8B, Supplementary Figures 6, 7). Remarkably, 90% of samples were similarly assigned to the same clusters across the two approaches, highlighting the high concordance between metaRNA-Seq and multi-omics integration. To validate the robustness of these clusters, we observed significant effect sizes for the independent compositions of the virome (*R*^2^ = 0.58, *p* = 0.004 for both multi-omics and metaRNA-Seq) and the transcriptome (*R*^2^ = 0.92, *p* = 0.001 for multi-omics; *R*^2^ = 0.90, *p* = 0.003 for metaRNA-Seq), whereas only the bacteriome composition from 16S-Seq showed a significant association with the clustering (*R*^2^ = 0.35, *p* = 0.044) (Figure 8C).

We then assessed the biological relevance of these clusters. Functional enrichment analysis of host transcripts revealed two pathways significantly differing between clusters (autophagosome maturation and nucleus organization), while many immune-related pathways were consistently enriched across both datasets (Figure 8D). Specifically, for both datasets, Cluster C1 was characterized by signatures of mRNA and rRNA metabolism, whereas Cluster C2 displayed an inflammatory transcriptional profile enriched in pathways related to myeloid activation, phagocytosis, neutrophil chemotaxis, and cell surface signaling.

Interestingly, while no microbiome features were significantly associated with the two clusters in the multi-omics analysis, metaRNA-Seq revealed distinct microbial signatures. The inflammatory cluster C2 was notably associated with transcriptional activity from *Streptococcus pneumoniae*, whereas Cluster C1 was enriched in RNA from *Streptococcaceae* phages, greater richness of transcriptionally active bacterial taxa, and higher transcriptionally active prokaryotic DNA phage diversity. These findings suggest that metaRNA-Seq alone is capable of reproducing integrative multi-omics clustering, while also offering greater sensitivity in capturing active microbial components, particularly at the species and phage level. This positions metaRNA-Seq as a powerful, single-assay alternative for exploring host–microbiome endotypes in complex microbial ecosystems. Despite the limited sample size, we tested whether the cluster assignments derived from metaRNA-Seq were associated with clinical features (Supplementary Table S6). We observed that the inflammatory/pneumococcal C2 cluster had a higher rate of ventilation requirement (41.7% vs. 12.5%), although no clinical or biological feature reached statistical significance.

## Discussion

The present study aimed to assess the ability of metatranscriptomics to comprehensively characterize the nasopharyngeal microbiome compared with kingdom-specific sequencing techniques. By comparing the virome, bacteriome, and human coding transcriptome of 20 nasopharyngeal samples from infants under 7 months of age hospitalized for bronchiolitis, we found that metatranscriptomics showed a good agreement with single-kingdom approaches for RNA viruses and the host-transcriptional response, such as the antiviral response. Interestingly, RNA from eukaryotic and prokaryotic DNA viruses was also detected, which suggested transcriptional activity in their infected hosts. Indeed, our vir-mNGS protocol detects extracellular viral particles, while metaRNA-Seq captures total viral RNA (such as intracellular transcripts and extracellular RNA viruses). Thus, DNA viruses with RNA reads suggest active transcription, whereas those without RNA would likely represent non-replicating forms, such as extracellular bacteriophages. It is also plausible that a proportion of the detected viral DNA derives from prophages integrated within residual bacteria that persisted despite the pretreatment procedures. These observations are in agreement with previous studies that identified DNA bacteriophages with RNA-Seq protocols (Rajagopala et al., 2021; Santiago-Rodriguez et al., 2015; Wang et al., 2023). However, further studies are needed to investigate whether the phage lifestyle (lytic or lysogenic) could be identified by analyzing specific transcripts, such as integrins or lysins (Santiago-Rodriguez et al., 2015). Likewise, a better understanding is needed to assess extent to which metatranscriptomics may differentiate latent from replicative eukaryotic DNA viruses such as *Herpesviridae*, and how this feature would impact patients’ management.

Additionally, metaRNA-Seq highlighted different transcriptional activity patterns of bacterial genera in the nasopharynx of children suffering from severe bronchiolitis. In addition to the fact that metaRNA-Seq fine-tuned the profile of the active bacteriome down to species identification, and while 16S profiling generally informs about bacteria presence, with sometimes poor species resolution for short-reads sequencing (Jovel et al., 2016; Johnson et al., 2019), metaRNA-Seq showed that *Streptococcus* and *Haemophilus* genera presented high transcriptional activity compared to *Moraxella* and *Staphylococcus* genera. This result is in concordance with the known associations between severe bronchiolitis and *Streptococcus* or *Haemophilus* genus coinfections (Man et al., 2017; de Steenhuijsen Piters et al., 2020) as these genera were associated with a high bacterial transcriptional activity even when bacteria were poorly abundant. Furthermore, metatranscriptomics cross-kingdom integration showed similar performances compared with multi-omics integration. This integration enabled the identification of a more “inflammatory” profile, which was associated with RNA of *Streptococcus pneumoniae* and inversely associated with the transcriptional activity of *Streptococcaceae* phage. This hyperinflammatory profile associated with RNA from respiratory microorganisms has also been reported in other studies, such as that of Zou and colleagues, which demonstrated that metatranscriptomics can distinguish LRTI from non-LRTI cases in adults based on integrated host–microbial signatures (Zou et al., 2025). As the purpose of the study was primarily methodological, the sample size was limited due to the cost of the sequencing techniques. Therefore, associations between respiratory microbial–host endotypes and clinical features of young children with bronchiolitis should be further investigated in future studies.

Such methodological comparison is also determinant for identifying contaminants and false positive identifications in metaRNA-Seq and removing them efficiently to not bias interpretation (Goswami et al., 2021). These false positive identifications could originate from misclassification of human reads or contaminated references in the database (Sun et al., 2023). In the present study, we frequently noted the presence of viruses belonging to the *Nairoviridae* family in metaRNA-Seq results, which are unlikely to be found in infant nasopharyngeal samples. Other metaRNA-Seq specific identifications represented viral and bacterial species that could either be true identification with low abundance or specific contaminants not efficiently removed in our process (Sabatier et al., 2020; Shaffer et al., 2022; Elie et al., 2023). Discordances between metaRNA-Seq and 16S sequencing may primarily reflect technical limitations. Indeed, the majority (82.8%) of taxa missed by 16S sequencing were present at very low relative abundances in metaRNA-Seq, likely falling below the sensitivity of 16S or competing with diverse bacterial DNA. Similarly, 90.6% bacterial genera detected by 16S but absent in metaRNA-Seq often had less than 0.5% relative abundance, reflecting insufficient sequencing depth to capture low-transcriptionally active bacteria. Nevertheless, some genera (*Actinobacillus*, *Bergeyella*, *Alloprevotella*, and *Granulicatella*), sometimes at higher relative abundances, were consistently found in multiple samples by 16S yet never detected in metaRNA-Seq. This observation may reflect differences in biological activity, with bacterial DNA present but transcripts absent or expressed at very low levels. Such high DNA/RNA discordances may indicate low-transcriptional activity or inactivity like dead bacteria, potentially influenced by interactions with host-immunity or virulent bacteriophages. Future studies comparing DNA and RNA levels with bacterial culture capacities could clarify whether metatranscriptomics effectively distinguishes active from inactive bacteria. Of note, extractions platforms were different between metaRNA-Seq and targeted-kingdom techniques and may have influenced the results. Furthermore, a bias can also exist due to the additional cycle of thawing for metaRNA-Seq, although conservative RNA later was present, thus limiting RNA degradation.

Overall, our study highlights the possibility of interpreting several microbiome kingdoms from metatranscriptomics, when specific thresholds of sequencing depths are encountered. First, we observed that the plateau of the rarefaction curve was reached in all but one sample for bacterial metaRNA-Seq. This illustrates that sufficient depth (>1,000 bacterial reads) was globally obtained even in samples with low RNA bacterial relative abundance; nonetheless, making sure that the rarefaction curve reached is highly recommended. Second, as proposed in other studies (Gaston et al., 2022; Gu et al., 2016), we showed that using an internal RNA control could ensure a minimal detection threshold for viral metatranscriptomics. We chose an MS2 phage represented by a small genome of 2,800 pb length and spiked at a limit of detection, speculating that longer genomes present at the same concentration would be detected with more reads, yielding positive ALR. For negative ALR results, PCR testing should be considered for confirmation. We did not analyze bacterial results according to ALR normalization, as IC was not added for 16S-Seq, but we envisage that the use of a common IC for metaRNA-Seq could help to integrate results of the different kingdom views. Overall, a minimum of 1,000 bacterial reads and 100,000 human reads was required for reliable bacterial and human transcriptome profiling, respectively, while the inclusion and detection of an internal control was essential for the identification of low-abundance viral signals. Given the variability in component proportions between samples, defining a universal minimal sequencing depth remains challenging. In this study, interpretable results were consistently obtained for samples exceeding 15.6 million raw reads. The general recommendation of 20–50 million reads seems reasonable for providing comprehensive profiling, interpreting the human response, and detecting rare microorganisms. Other optimizing strategies are also possible to go around this situation. Depletion of bacterial 16S rRNA or unwanted human reads by specific probes or CRISPR/Cas9-derived methods may increase sequencing depth (Prezza et al., 2020; Wahl et al., 2022). Moreover, RNA extraction combining mechanical and chemical lysis is crucial (Michel et al., 2025).

From a methodological standpoint, the study presents some limitations. First, the small sample size may have hindered the evaluation of metatranscriptomics performances in more variable microbiome contexts. Second, the study focused only on nasopharyngeal samples from infants suffering from bronchiolitis. The performances of metatranscriptomics should also be evaluated across different microbial biomasses and clinical contexts. Third, we used Kraken2, a rapid and recommended taxonomy classifier for read assignment (Wood et al., 2019). Other methods, such as *de novo* reconstruction of longer contigs into Metagenome-associated Genomes (MAGs), could further enhance the accuracy of taxonomic profiling (Yang et al., 2021). Nonetheless, several benchmarks evaluating taxonomic profilers have identified Kraken2 as a powerful tool for eukaryotic viruses and bacteriophages, as well as for bacteria assignment (Wood et al., 2019; Ye et al., 2019; Glickman et al., 2021). It is worth noting that these benchmarks were primarily conducted on metagenomics data, but recent studies have begun focusing on critical dry-lab steps for metatranscriptomics, such as taxonomic profiling, sequence alignment, and differential gene expression analyses, to optimize data quality and interpretation (Cho et al., 2023; Li et al., 2025). Read-level decontamination could further improve confidence in distinguishing contaminants but requires specific development and validation. Moreover, future validation using standardized mock communities will be valuable to better distinguish method-related technical sensitivity from genuine biological signals. Importantly, we did not include DNA shotgun metagenomics for the comparison. This latter technique is less biased regarding 16S rDNA copy number variations among bacteria. Furthermore, in the microbiome field, shotgun metagenomics is classically used to provide taxonomic classification of the microbiome down to the species (Johnson et al., 2019) and even to strain levels (Anyansi et al., 2020). Metatranscriptomics can provide both identification of active bacteria down to the species level and potentially identify functional pathways under transcription (Abu-Ali et al., 2018; Korry et al., 2020). However, analyses, such as metabolism activity or resistome profiling, were not explored in this study. Fungal metatranscriptomics was not investigated as our extraction methods were not suited for fungi extraction. Finally, from a clinical perspective, this study did not allow for definitive conclusions regarding physiopathology and disease-associated signatures. Future studies should increase the sample size, include more recent winters, incorporate multicenter cohorts, and consider healthy pediatric controls.

Nonetheless, this study demonstrates that metatranscriptomics represents a powerful tool for pinpointing active microorganisms in acute respiratory disease, along with host-response exploration. This study joins the few studies that have compared microbial identification by metatranscriptomics with clinical routine tests (Ojala et al., 2023; Langelier et al., 2018; Cunningham-Oakes, 2023; Nicolas et al., 2023; Twin et al., 2013; Doxey et al., 2025; Wright et al., 2024). Its main advantage lies in enabling simultaneous observation of multiple layers of the active microbiome in a single workflow, facilitating inter-kingdom analyses. In practice, metatranscriptomics can be used as a first-line approach when the main pathogen is already known but information on microbial activity and host response is required; when the pathogen is unknown and a broad capture of viral RNAs is needed, as in cases of emerging or novel RNA viruses; or when diagnostic uncertainty warrants screening for the most transcriptionally active microorganisms. Conversely, kingdom-specific or enrichment-based methods remain essential when very low-level pathogens must be detected or when the DNA microbiome needs to be characterized to explore the microbial reservoir. In our estimation, the reagent cost of metatranscriptomics represents approximately three-quarters of that required for a combined multi-omics approach. Beyond its potential implications on therapeutic adaptation and clinical management, particularly in cases of undocumented infections, metatranscriptomics should also be further evaluated through health economic studies, considering both its technical advantages and its potential to reduce hospitalization-related costs. In the era of metaomics advances, combining enriched RNA sequences of interest, automated processes, real-time long reads sequencing with sufficient depth, and robust computational analyses might improve the rapidity of metatranscriptomics at an affordable price in the (near) future (Chanderraj and Dickson, 2018).

## Conclusion

Overall, we present a comparison of sequencing methods using clinical respiratory samples to evaluate the concordance and complementarity of kingdom-specific and metatranscriptomic approaches. We provide practical guidelines for the use and interpretation of metatranscriptomics in cross-kingdom data integration, which will help advance understanding of the role of the respiratory microbiome in health and disease.

## Data Availability

The datasets presented in this study can be found in online repositories. The names of the repository/repositories and accession number(s) can be found at: https://www.ncbi.nlm.nih.gov/, PRJNA1076126.

## Footnotes

1
https://rvdb.dbi.udel.edu/previous-release
