# Supplementary material for: Comparison between metatranscriptomics and viral metagenomics, 16S, and host transcriptomics for comprehensive profiling of the respiratory microbiome and host response
Source: Front Microbiol. 2026 Jan 7;16:1685035. doi: 10.3389/fmicb.2025.1685035 (PMC12819617; doi:10.3389/fmicb.2025.1685035)
Supplement: Supplementary file 11 [file Data_Sheet_1.docx]

**Supplementary materials**


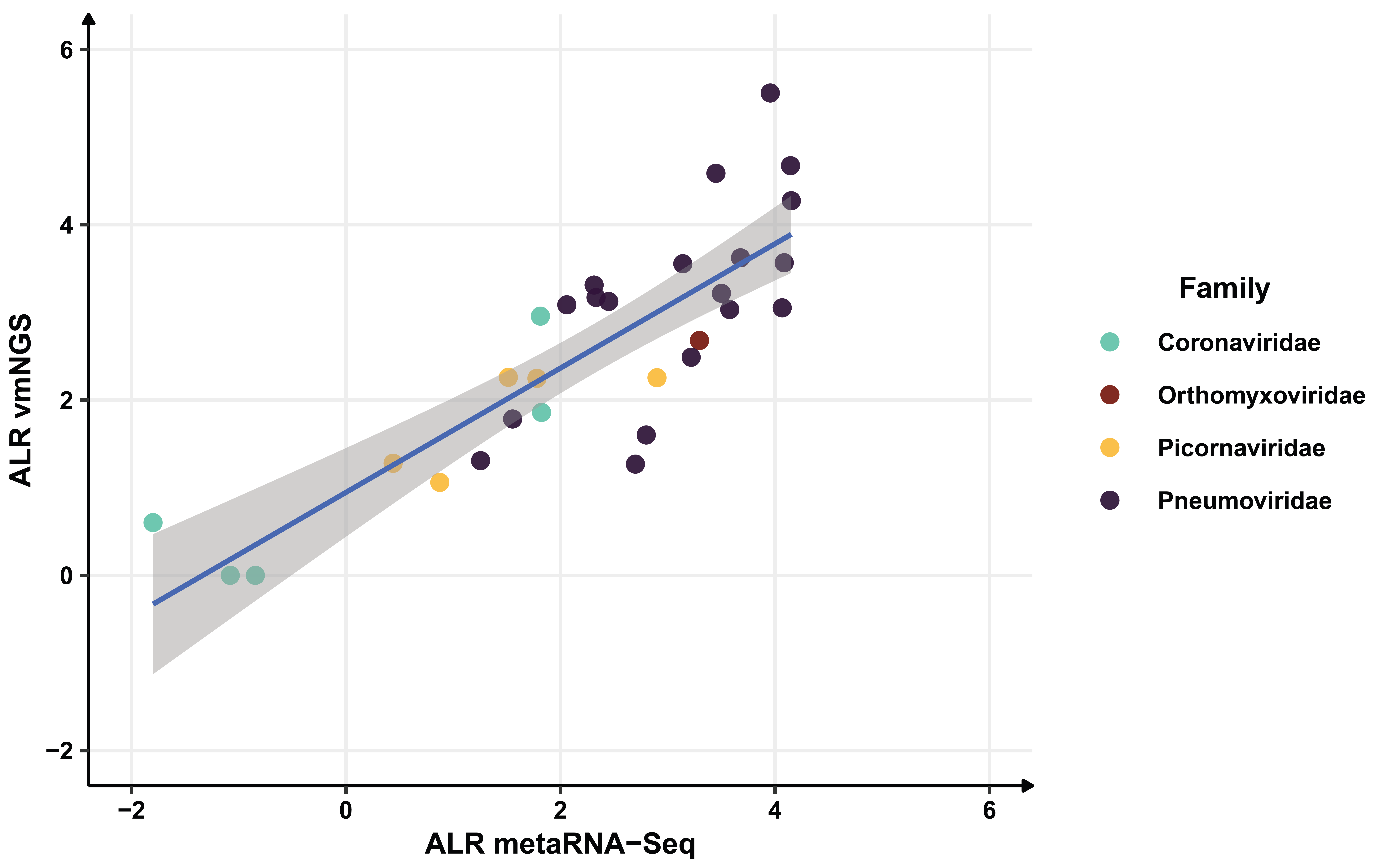


**Figure S1. Correlation of RNA virus expression between viral metagenomics (vmNGS) and viral metatranscriptomics (metaRNA-Seq).** Each color represents a viral family. The 31 viral hits detected in the 20 samples by both techniques are expressed as additive log-ratios (ALR) using MS2 reads as the reference denominator. The blue line represents the regression curve (p<0.001, Pearson r=0.83), with the gray area indicating the 95% confidence interval.


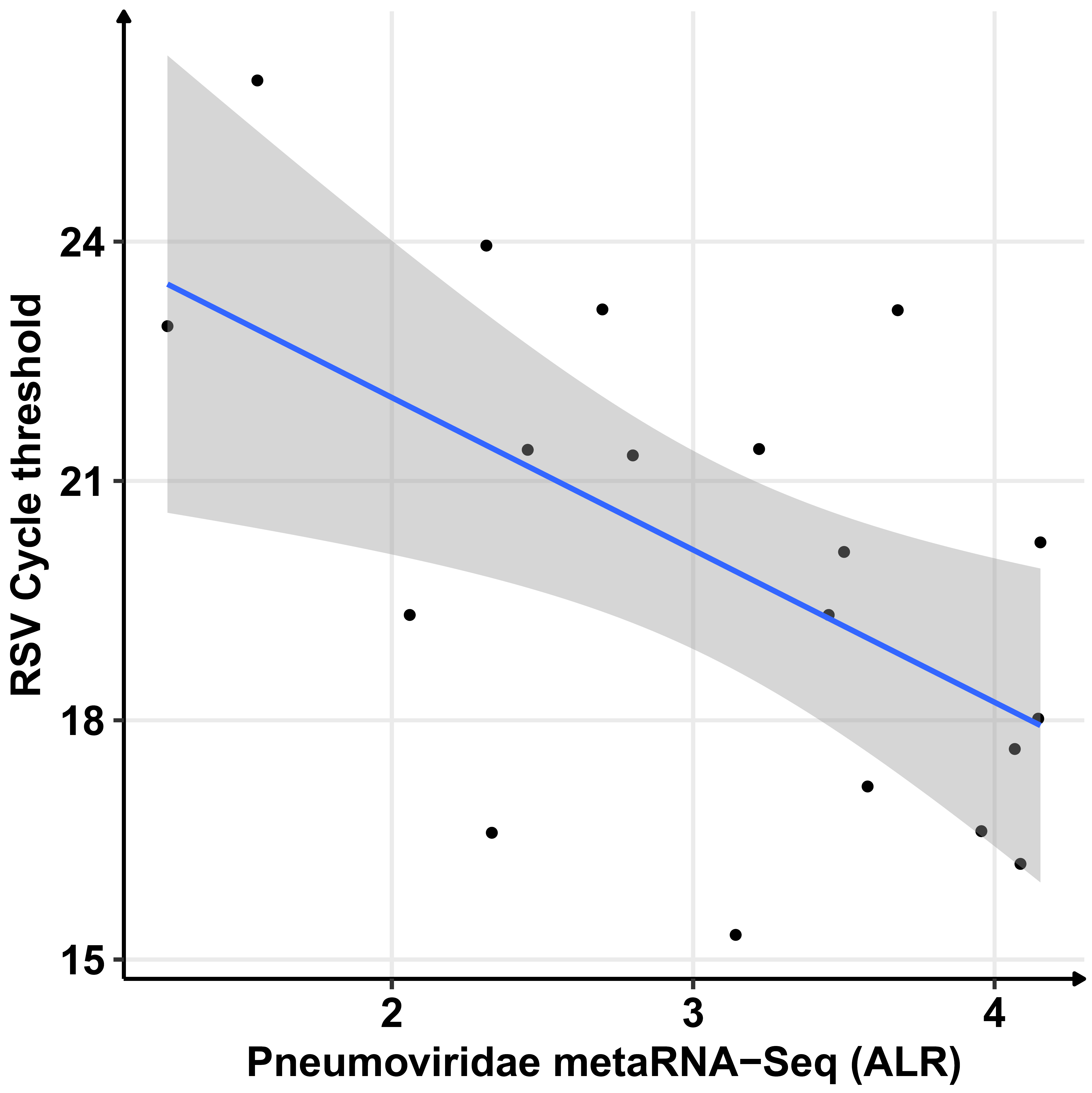


**Figure S2. Correlation of Respiratory Syncitial Virus (RSV) quantification between RT-PCR and viral metatranscriptomics (metaRNA-Seq).** In the 19 RSC-positive samples, cycle threshold values are plotted against the expression of *Pneumoviridae* hits detected by metaRNA-Seq and expressed as additive log-ratios (ALR) using MS2 reads as the reference denominator. The blue line represents the correlation curve (Pearson r=-0.6, p=0.03), with the gray area indicating the 95% confidence interval.

**Figure S3. Bacterial genera detected exclusively by 16S-Seq.** Bar plots (left panel) indicate the number of positive samples, and box plots (right panel) show the corresponding distribution of relative abundances, with medians represented, for bacterial genera identified solely by 16S-Seq.

**Figure S4. Bacterial genera detected exclusively by metaRNA-Seq.**  Bar plots (left panel) indicate the number of positive samples, and box plots (right panel) show the corresponding distribution of relative abundances, with medians represented, for bacterial genera identified solely by metaRNA-Seq.

A B


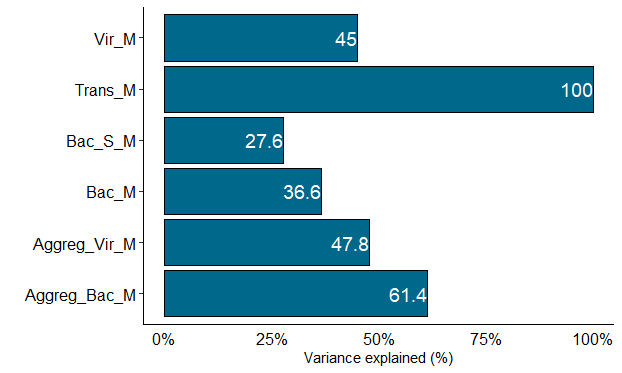

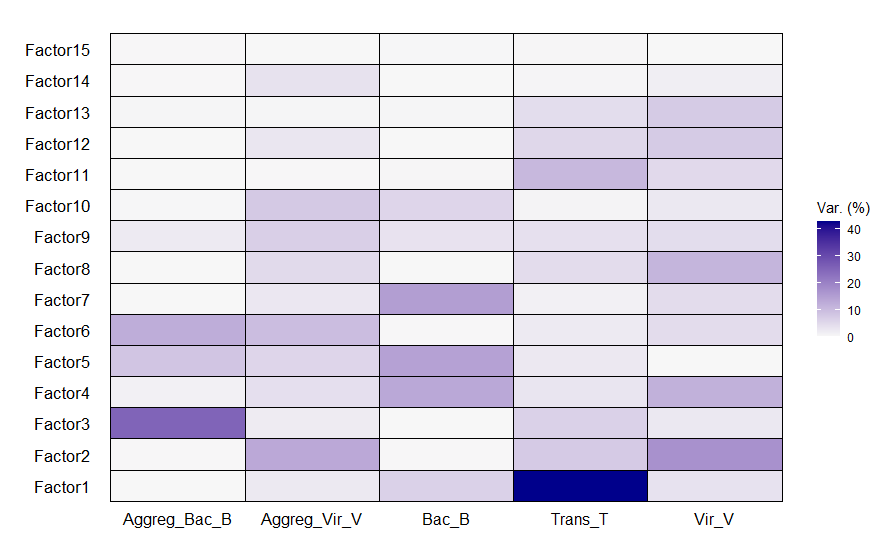

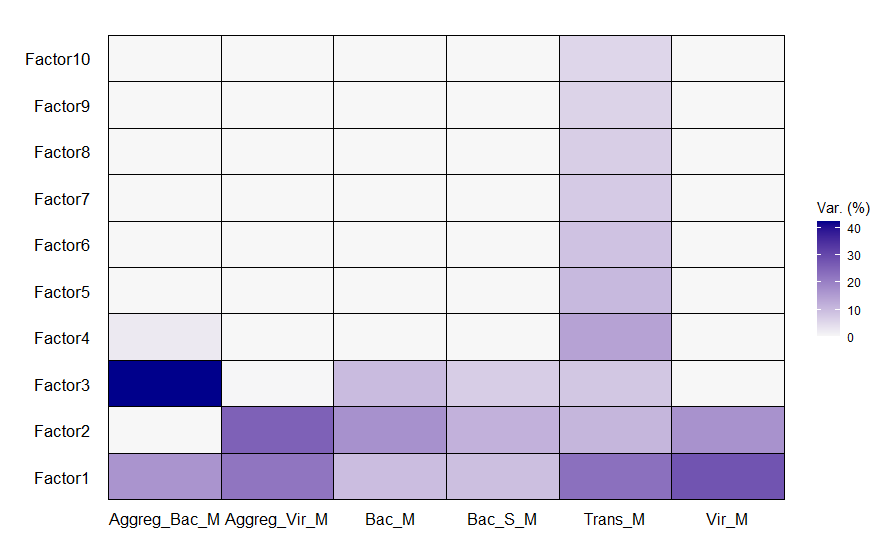

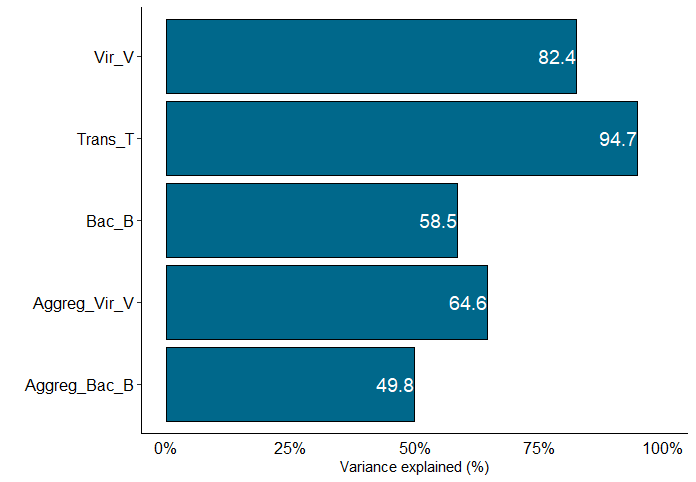


**Figure S5. Results of Multi-Omics Factor Analysis (MOFA) for kingdom-specific multi-omics and metatranscriptomcis (metaRNA-Seq) approaches.** For each MOFA model, the upper panel represents a heatmap showing the contribution of data view to the variance of individual latent factors identified in the integrated model, constructed from multi-omics (A) or metaRNA-Seq (B) datasets. The lower panel displays the total variance explained by each view across the overall model.

*Abreviations of the views:* *Vir_V: Virome (v-mNGS); Trans_T: Host Transcriptome (mRNA-Seq); Bac_B: Bacteriome (16S-Seq); Aggreg_Vir_V: Aggregated data for virome obtained with vm-NGS (e.g; viral load; Shannon and richness); Aggreg_Bac_B: Aggregated data for bacteriome obtained by 16S-Seq (e.g; DNA bacterial load; Shannon; and richness). Vir_M: Virome (metaRNA-Seq); Trans_M: Host Transcriptome (metaRNA-Seq); Bac_B: Bacteriome (metaRNA-Seq); Aggreg_Vir_V: Aggregated data for virome obtained with metaRNA-Seq (e.g; viral load; Shannon and richness); Aggreg_Bac_B: Aggregated data for bacteriome obtained by metaRNA-Seq (e.g; RNA bacterial load; Shannon; and richness).*

A


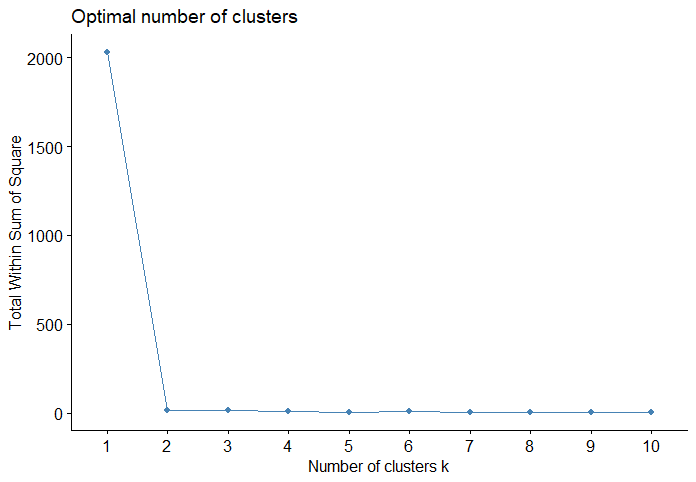

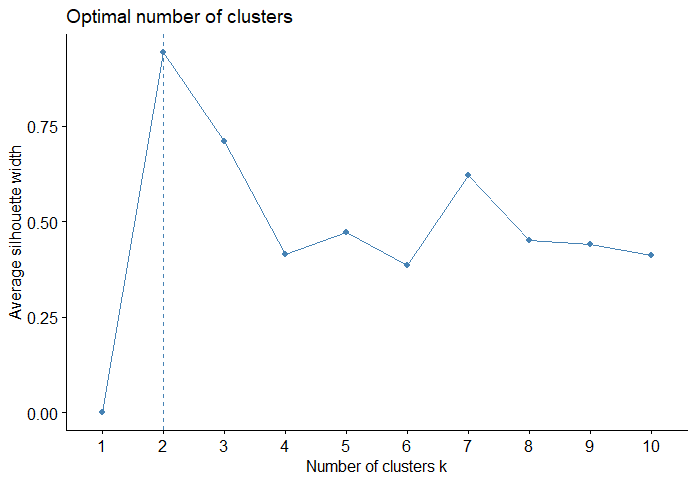

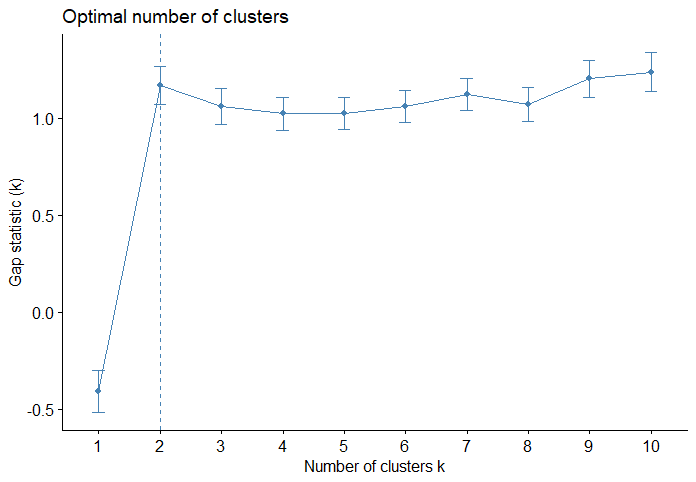

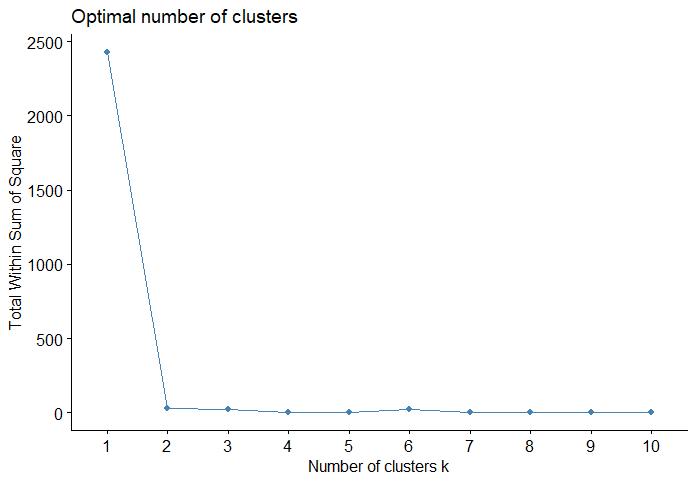

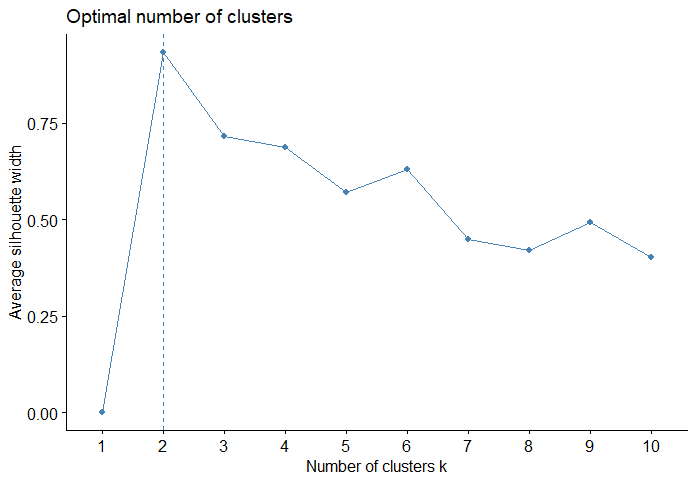

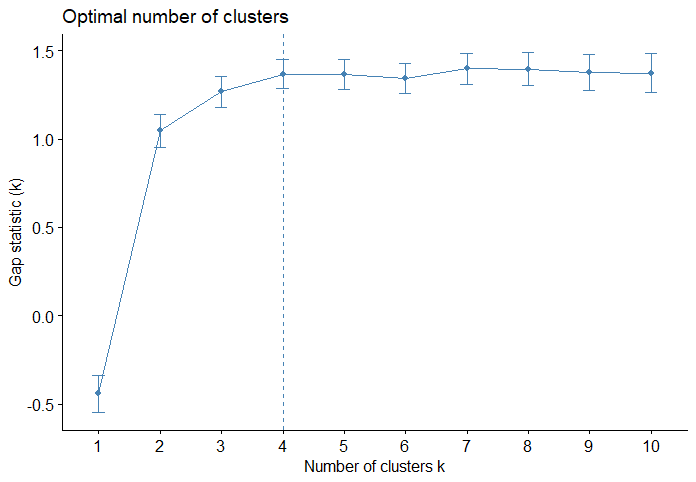


B

**Figure S6. Determination of the optimal number of integrated microbial-host clusters** The optimal number of clusters for kingdom-specific multi-omics (A) and metatranscriptomics (metaRNA-Seq) (B) datasets was determined using Uniform Manifold Approximation Projection (UMAP) embeddings derived from the latent factors of each MOFA model. Three clustering indices were used to guide this determination, shown from left to right): total within-cluster sum of squares; silhouette width; and gap statistic.


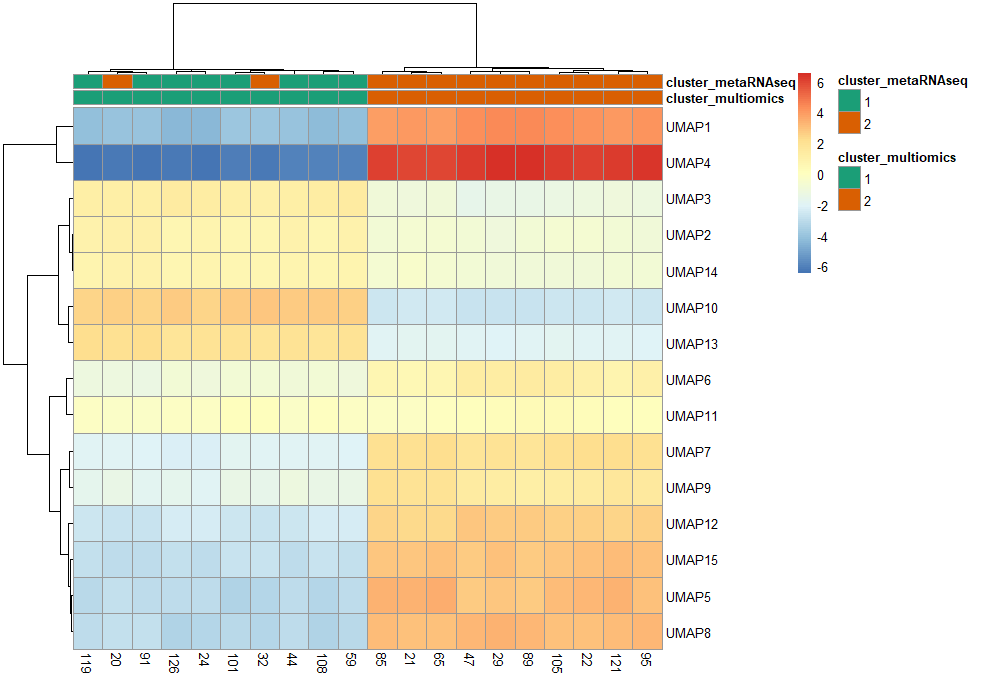


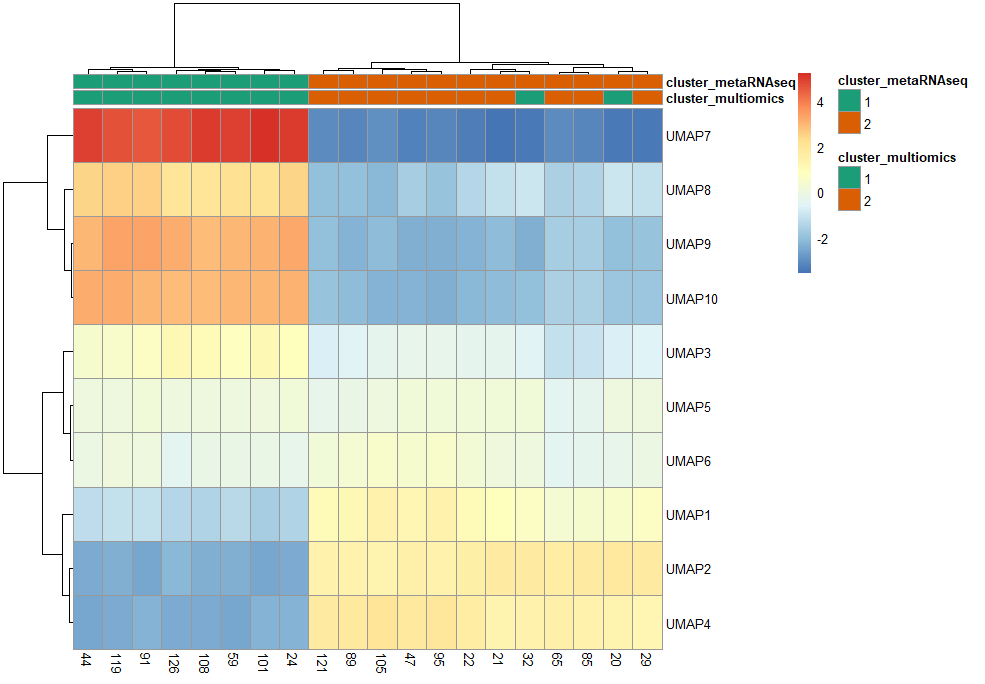


Figure S7: Heatmaps of the UMAP dimensions used for clustering in the multi-omics dataset (upper heatmap) and the metaRNA-Seq dataset (lower heatmap). The annotated columns indicate the distribution of samples across the two clusters (shown in dark and orange) identified by the k-means method for each dataset.
